# Under an integrative taxonomic approach: the description of a new species of the genus *Loxosceles* (Araneae, Sicariidae) from Mexico City

**DOI:** 10.3897/zookeys.892.39558

**Published:** 2019-11-27

**Authors:** Alejandro Valdez-Mondragón, Claudia I. Navarro-Rodríguez, Karen P. Solís-Catalán, Mayra R. Cortez-Roldán, Alma R. Juárez-Sánchez

**Affiliations:** 1 CONACYT Research Fellow. Laboratory of Arachnology (LATLAX), Laboratorio Regional de Biodiversidad y Cultivo de Tejidos Vegetales (LBCTV), Instituto de Biología, Universidad Nacional Autónoma de México (UNAM), sede Tlaxcala, Ex-Fábrica San Manuel, San Miguel Contla, C. P. 90640 Santa Cruz Tlaxcala, Tlaxcala, Mexico; 2 Laboratory of Arachnology (LATLAX), Laboratorio Regional de Biodiversidad y Cultivo de Tejidos Vegetales (LBCTV), Instituto de Biología, Universidad Nacional Autónoma de México (UNAM), sede Tlaxcala, Mexico; 3 Colección Nacional de Arácnidos (CNAN), Departamento de Zoología, Instituto de Biología, Universidad Nacional Autónoma de México, (UNAM), Ciudad Universitaria, Apartado Postal 04510, Coyoacán, Mexico City, Mexico; 4 Posgrado en Ciencias Biológicas, Centro Tlaxcala de Biología de la Conducta (CTBC), Universidad Autónoma de Tlaxcala (UATx), Carretera Federal Tlaxcala-Puebla, Km. 1.5, C. P. 90062, Tlaxcala, Tlaxcala, Mexico

**Keywords:** DNA barcodes, ecological niche modeling, *Loxosceles tenochtitlan* sp. nov., species delimitation, taxonomy

## Abstract

A new species of the spider genus *Loxosceles* Heineken & Lowe, 1832, *Loxosceles tenochtitlan* Valdez-Mondragón & Navarro-Rodríguez, **sp. nov.**, is described based on adult male and female specimens from the states of Mexico City, Estado de Mexico and Tlaxcala. Integrative taxonomy including traditional morphology, geometric and lineal morphology, and molecules (DNA barcodes of cytochrome *c* oxidase subunit 1 (CO1) and internal transcribed spacer 2 (ITS2)), were used as evidence to delimit the new species. Four methods were used for molecular analyses and species delimitation: 1) corrected *p*-distances under neighbor joining (NJ), 2) automatic barcode gap discovery (ABGD), 3) general mixed yule coalescent model (GMYC), and 4) poisson tree processes (bPTP). All molecular methods, traditional, geometric and lineal morphology were consistent in delimiting and recognizing the new species. *Loxosceles tenochtitlan***sp. nov.** is closely related to *L. misteca* based on molecular data. Although both species are morphologically similar, the average *p*-distance from CO1 data was 13.8% and 4.2% for ITS2 data. The molecular species delimitation methods recovered well-supported monophyletic clusters for samples of *L. tenochtitlan***sp. nov.** from Mexico City + Tlaxcala and for samples of *L. misteca* from Guerrero. *Loxosceles tenochtitlan***sp. nov.** is considered a unique species for three reasons: (1) it can be distinguished by morphological characters (genitalic and somatic); (2) the four different molecular species delimitation methods were congruent to separate both species; and (3) there is variation in leg I length of males between both species, with the males of *L. misteca* having longer legs than males of *L. tenochtitlan***sp. nov.**, also morphometrically, the shape of tibiae of the palp between males of both species is different.

## Introduction

The spider family Sicariidae Keyserling, 1880 comprises three genera: *Hexophthalma* Karsch, 1879 with eight species from Africa, *Sicarius* Walckenaer, 1847 with 21 species distributed in Central and South America, and *Loxosceles* Heineken & Lowe, 1832, with 139 described species worldwide (Magalhães et al. 2017; WSC 2019). Spiders of the genus *Loxosceles* are better known in North America as “violin spiders”, “recluse spiders”, or “brown recluse spiders”. They are well known by the medical community and general public as their bites can cause dermonecrotic lesions due to the action of Sphingomyelinase D, an enzyme in their venom that destroys endothelial cells lining the blood vessels (Sandidge and Hopwood 2005; Vetter 2008, 2015; Ramos-Rodríguez and Méndez 2008; Manríquez and Silva 2009; Swanson and Vetter 2009).

Gertsch (1958, 1967) and Gertsch and Ennik (1983) proposed that the species of *Loxosceles* belong in eight species groups: *reclusa, laeta, amazonica, gaucho, spadicea, rufescens, vonwredei*, and *spinulosa*. However, Duncan et al. (2010) and Fukushima et al. (2017), using molecular data, synonymized the species group *amazonica* with the species group *rufescens*; therefore, the genus is currently composed of seven species groups. The *reclusa* group has the highest diversity, with more than 50 species from North America, the majority from Mexico (Gertsch and Ennik 1983).

Mexico has the highest diversity of *Loxosceles* worldwide, with 39 species, 37 native (not including the new species described here), and two introduced species: *Loxosceles reclusa* Gertsch & Mulaik, 1940 and *Loxosceles rufescens* (Dufour, 1820) (Gertsch 1958, 1973; Gertsch and Ennik 1983; Valdez-Mondragón et al. 2018b, WSC 2019). Species diversity is greater in the north, decreasing to the south (Valdez-Mondragón et al. 2018b: figs 73–76). The states with the greatest diversity are Baja California Sur, Baja California and Sonora, with five species each (Valdez-Mondragón et al. 2018a, b). *Loxosceles boneti* Gertsch, 1958 is the most common species in Mexico, primarily found in the central region of the country (Valdez-Mondragón et al. 2018b). The preferred habitats of *Loxosceles* in Mexico are mainly dry and tropical forests, including tropical deciduous forests, and deserts; however, some species, such as *L. chinateca* and *L. yucatana*, are distributed in tropical rain forests. Additionally, some species have been recorded from caves, a preferred microhabitat of Mexican species (e.g., *L. misteca, L. boneti, L. chinateca, L. tehuana, L. tenango*, and *L. yucatana*) (Valdez-Mondragón et al. 2018b). The first species described from Mexico was *Loxosceles yucatana* Chamberlin & Ivie, 1938 from the Yucatan Peninsula, and the most recently described was *Loxosceles malintzi* Valdez-Mondragón, Cortez-Roldán, Juárez-Sánchez & Solís-Catalán, 2018 from the central region of Mexico (Valdez-Mondragón et al. 2018b). To date, the taxonomy of the species has been based only on traditional morphology, using genitalic characters, male palps and seminal receptacles in females.

Modern taxonomy uses multiple lines of evidence for species recognition, identification, diagnosis and delimitation. Several recently developed molecular delimitation methods have highlighted the extensive inconsistency in classical morphological taxonomy (Ortiz and Francke 2016). Molecular methods have provided a new way to resolve species delimitation problems by using the infra-specific genealogical information in DNA markers which provides objective implementation of modern species concepts (e.g., biological, phylogenetic, genotypic cluster). The appropriate way to species delimitation research is to analyze the data with a wide variety of methods and different lines of evidence to delimit lineages that are consistent across the results, understanding the behavior of the molecular species delimitation methods and contributing in this way to integrative taxonomy (Carstens et al. 2013, Luo et al. 2018).

Currently, there are two separate tasks to which DNA barcodes are being applied in modern systematics. The first is distinguishing between species (equivalent to species identification or species diagnosis), and the second is the use of DNA data to discover new species (equivalent to species delimitation and species description) (DeSalle et al. 2005). For some groups of organisms, including some groups of spiders, morphology alone cannot determine species boundaries, and identifying morphologically inseparable cryptic or sibling species requires a new set of taxonomic tools, including the analysis of molecular data (Jarman and Elliott 2000; Witt and Hebert 2000; Proudlove and Wood 2003; Hebert et al. 2003, 2004; Bickford et al. 2007; Hamilton et al. 2011, 2014, 2016; Ortiz and Francke 2016). The spider genus *Loxosceles* is no exception. Recent studies based on molecular evidence have suggested that the known diversity within the genus could be highly underestimated (Binford et al. 2008; Duncan et al. 2010; Planas and Ribera 2014, 2015; Tahami et al. 2017). One important factor leading to the underestimation is widespread intraspecific variation in sexual structures, mainly in the seminal receptacles of females, something noted previously by Brignoli (1968) and Gertsch and Ennik (1983) and recently by Valdez-Mondragón et al. (2018b) in the case of the species from Mexico.

The primary aim of this study is to use an integrative taxonomic approach for the delimitation and description of a new species of *Loxosceles* from Mexico City. We analyzed DNA barcodes and used traditional morphology, ultra-morphology, geometric and linear morphometrics, biogeography, and ecological niche modeling for species delimitation. This is the first-time multiple lines of evidence have been used in the taxonomy of the genus.

## Materials and methods

### Biological material

The specimens of the new species were collected and deposited in 80% ethanol and labeled with their complete field data. The type specimens and additional examined material are deposited with their collection codes in the Laboratory of Arachnology (**LATLAX**), Laboratorio Regional de Biodiversidad y Cultivo de Tejidos Vegetales (**LBCTV**), Institute of Biology, Universidad Nacional Autónoma de México (**IBUNAM**), Tlaxcala City. The male holotype of *Loxosceles misteca* Gertsch, 1958 was examined and is deposited at the American Museum of Natural History (**AMNH**). The descriptions and observations of the specimens were done using a Zeiss Discovery V8 stereoscope. A Zeiss Axiocam 506 color camera attached to a Zeiss AXIO Zoom V16 stereoscope was used to photograph the different structures of specimens. The female seminal receptacles and male palps were dissected in ethanol (80%) and cleaned in potassium hydroxide (KOH-10%) for 5 to 10 min. Habitus, seminal receptacles and palps were submerged in 96% gel alcohol and covered with a thin layer of liquid ethanol (80%) to minimize diffraction during photography (Valdez-Mondragón and Francke 2015). For the photomicrographs, the morphological structures were dissected and cleaned with an ultrasonic cleaner at 20–40 kHz; subsequently, they were critical-point dried, and examined at low vacuum in a Hitachi S-2460N Scanning Electron Microscope (**SEM**). The descriptions follow Valdez-Mondragón et al. (2018b). All measurements in the descriptions are in millimeters (mm). Scale measurements on photomicrographs are in micrometers (μm). The distribution map was made using QGIS v. 2.18. Expeditions for collecting additional material deposited at LATLAX of different species were carried out in Puebla (March and June 2017), Tlaxcala (April 2017), Hidalgo (May 2017), Oaxaca (June 2017), Guerrero (September 2017), and Oaxaca (March 2018). Abbreviations:

**AME** anterior median eyes;

**PLE** posterior lateral eyes;

**PLS** posterior lateral spinnerets;

**PME** posterior median eyes.

### Taxon sampling

The molecular analyses presented here are based on a total of 52 individuals from 11 species of *Loxosceles*, including the new species described here and two outgroups: *Loxosceles rufescens* (Dufour, 1820) and *Scytodes thoracica* (Latreille, 1802) (Table 1). Three different partitions were used (CO1: 656 bp, ITS: 435 bp, and CO1+ITS2: 1091 bp).

**Table 1.** Specimens sequenced for each species, DNA voucher numbers, localities, and GenBank accession numbers.

Species	DNA voucher LATLAX	Locality	GenBank accession number	
			CO1	ITS2
*L. misteca*	Ara0082	Mexico: Guerrero	MK936272	MK957212
	Ara0089	Mexico: Guerrero	MK936273	MK957215
	Ara0090	Mexico: Guerrero	MK936274	MK957214
	Ara0084	Mexico: Guerrero	MK936275	MK957213
	Ara0236	Mexico: Guerrero	MK936276	–
	Ara0237	Mexico: Guerrero	MK936277	–
***L. tenochtitlan* sp. nov.**	Ara0146	Mexico: Mexico City	MK936278	MK957209
	Ara0161	Mexico: Mexico City	MK936279	–
	Ara0173	Mexico: Tlaxcala	MK936280	MK957210
	Ara0164	Mexico: Tlaxcala	MK936281	MK957211
*L. malintzi*	Ara0100	Mexico: Guerrero	MK936282	MK957220
	Ara0001	Mexico: Puebla	MK936283	MK957218
	Ara0002	Mexico: Puebla	MK936284	–
	Ara0025	Mexico: Puebla	MK936285	MK957219
	Ara0072	Mexico: Puebla	MK936286	MK957222
	Ara0074	Mexico: Puebla	MK936287	MK957223
	Ara0101	Mexico: Guerrero	MK936288	–
	Ara0004	Mexico: Puebla	MK936289	MK957221
*L. tenango*	Ara0191	Mexico: Hidalgo	MK936290	–
	Ara0192	Mexico: Hidalgo	MK936291	MK957201
	Ara0045	Mexico: Hidalgo	–	MK957195
	Ara0189	Mexico: Hidalgo	–	MK957196
	Ara0190	Mexico: Hidalgo	–	MK957197
	Ara0193	Mexico: Hidalgo	–	MK957198
	Ara0188	Mexico: Hidalgo	–	MK957200
*L. jaca*	Ara0186	Mexico: Hidalgo	MK936292	MK957194
	Ara0048	Mexico: Hidalgo	MK936293	–
	Ara0046	Mexico: Hidalgo	–	MK957192
	Ara0047	Mexico: Hidalgo	–	MK957193
	Ara0183	Mexico: Hidalgo	–	MK957199
***Loxosceles* sp. 1**	Ara0175	Mexico: Hidalgo	MK936294	MK957208
	Ara0181	Mexico: Hidalgo	MK936295	MK957206
	Ara0182	Mexico: Hidalgo	MK936296	MK957207
	Ara0174	Mexico: Hidalgo	–	MK957202
	Ara0176	Mexico: Hidalgo	–	MK957203
	Ara0177	Mexico: Hidalgo	–	MK957204
	Ara0178	Mexico: Hidalgo	–	MK957205
*L. nahuana*	Ara0076	Mexico: Hidalgo	MK936297	MK957216
	Ara0077	Mexico: Hidalgo	MK936298	–
	Ara0079	Mexico: Hidalgo	MK936299	MK957217
*L. zapoteca*	Ara0094	Mexico: Guerrero	MK936300	MK957224
	Ara0220	Mexico: Guerrero	MK936301	–
	Ara0227	Mexico: Guerrero	MK936302	–
*L. colima*	Ara0115	Mexico: Colima	MK936303	MK957224
***Loxosceles* sp. 2**	Ara0194	Mexico: Guerrero	MK936304	–
	Ara0198	Mexico: Guerrero	MK936305	–
	Ara0199	Mexico: Guerrero	MK936306	–
	Ara0205	Mexico: Guerrero	MK936307	–
	Ara0209	Mexico: Guerrero	MK936308	–
	Ara0210	Mexico: Guerrero	MK936309	–
	Ara0204	Mexico: Guerrero	MK936310	–
*L. rufescens*	GenBank	Greece: Peloponnese	-	KR864735
*Scytodes thoracica*	GenBank	Turkey: Antalya	KR864739	

### DNA extraction, amplification and sequencing

Specimens for DNA extraction were preserved in ethanol (96%) and kept at -20 °C. DNA was isolated from legs, prosoma or complete specimens in the case of immatures. DNA extractions were done using a Qiagen DNeasy Tissue Kit following the protocol of Valdez-Mondragón and Francke (2015). DNA fragments included approximately 650 bp of the cytochrome *c* oxidase subunit 1 (CO1) mitochondrial gene, and 435 bp of the Internal Transcribed Spacer 2 (ITS2) nuclear gene. The fragments were ampliﬁed using the primers in Table 2. Ampliﬁcations were carried out in a Veriti Applied-Biosystems 96 Well Thermal Cycler, in a total volume of 25 μL: 3 μl DNA, 8.7 μl H_2_O, 12.5 μl Multiplex PCR Kit of QIAGEN, 0.4 μl of each molecular marker (forward and reverse). The PCR program for CO1 was as follows: initial step 1 min at 95 °C; amplification 35 cycles of 30 sec at 95 °C (denaturation), 30 sec at 48 °C (annealing), 1 min at 72 °C (elongation), and final elongation 5 min at 72 °C. PCR program for ITS2 was as follows: initial step 3 min at 94 °C; amplification 40 cycles of 30 sec at 94 °C (denaturation), 1 min at 53 °C (annealing), 1 min at 72 °C (elongation), and final elongation 5 min at 72 °C. PCR products were checked to analyze length and purity on 1% agarose gels with a marker of 100 bp and purified directly using the QIAquick PCR Purification kit of QIAGEN. DNA extraction and ampliﬁcation were performed at the Molecular Laboratory at the Laboratorio Regional de Biodiversidad y Cultivo de Tejidos Vegetales (LBCTV), Institute of Biology, Universidad Nacional Autónoma de México (UNAM), Tlaxcala City. Sequencing was performed at the Molecular Laboratory at the Institute of Biology, UNAM, Mexico City. Sequencing of both strands (5'–3'and 3'–5') of PCR products were performed in a Sequencer Genetic Analyzer RUO Applied Biosystems Hitachi model 3750xL. Sequence data of CO1 and ITS2 are deposited in GenBank with accession numbers: MK936272–MK936310 for CO1 and MK957192–MK957225 for ITS2 (Table 1).

**Table 2.** Primers used for PCR.

Gene	Primer name	Primer sequence (5'–3')	Reference
CO1	LCO	GGT CAA CAA ATC ATA AAG ATA TTG G	Folmer et al. (1994)
	HCO	TAA ACT TCA GGG TGA CCA AAA AAT CA	
	LCO-JJ	CHA CWA AYC ATA AAG ATA TYG G	Astrin and Stueben (2008)
	HCO-JJ	AWA CTT CVG GRT GCV CAA ARA ATC A	
ITS2	5.8S	CAC GGG TCG ATG AAG AAC GC	Ji et al. (2003), Planas and Ribera (2014)
	CAS28sB1d	TTC TTT TCC TCC SCT TAY TRA TAT GCT TAA	

### DNA sequence alignment and editing

Sequences were edited with the programs BioEdit v. 7.0.5.3 (Hall 1999) and Geneious v. 10.2.3 (Kearse et al. 2012). Sequences were aligned online using the default gap opening penalty of 1.53 in MAFFT (Multiple sequence alignment based on Fast Fourier Transform) v. 7 (Katoh and Toh 2008) using the following alignment strategy: Auto (FFT-NS-2, FFTNS-i or L-INS-i; depending on data size). These aligned matrices were subsequently used in analyses.

### Molecular analyses, species delimitation and haplotypes networks

For molecular species delimitation four methods were used for analyzing the concatenated CO1+ITS2 matrix (1091 characters): 1) *p*-distances under neighbor joining (NJ) using MEGA v. 7.0, 2) automatic barcode gap discovery (ABGD) online version (Puillandre et al. 2012) using both uncorrected and K2P distance matrices. 3) general mixed yule coalescent model (GMYC) (Pons et al. 2006) using GMYC web server (https://species.h-its.org/gmyc/), and 4) Bayesian Poisson tree process (bPTP) (Zhang et al. 2013, Kapli et al. 2017) using web server (https://species.h-its.org/ptp/). The models of sequence evolution were selected using the Akaike information criterion (AIC) in jModelTest v. 2.1.10 (Posada and Buckley 2004). The models selected for CO1 for each partition block were: GTR+G+I (1^st^ and 2^nd^ codon positions) and GTR+G (3^rd^ position). The model selected for ITS2 was GTR+G. The bootstrap values in the NJ analysis were calculated with the following commands: Number of replicates = 1000, Bootstrap support values = 1000 (significant values ≥ 50%), Substitution type = nucleotide, Model = Kimura 2-parameter, Substitution to Include = d: transitions + transversions, Rates among Sites = Gamma distributed (G), missing data treatment = pairwise deletion, select codon position= 1^st^+2^nd^+3^rd^+Noncoding Sites. The approaches for DNA barcoding tree-based delimitation explicitly use the phylogenetic species concept. A starting tree is input with Maximum Likelihood (ML) using MEGA v. 7.0, and Bayesian inference (BI) using MrBayes v. 3.1.2 (Ronquist and Huelsenbeck 2003) were implemented, and the analysis recognizes monophyletic cluster by searching differential intra- and inter-specific branching patterns (Ortiz and Francke 2016). The ML analysis was calculated with the parameters for CO1 and ITS2: Number of replicates = 1000, Bootstrap support values = 1000 (significant values ≥ 50%), Models of sequence evolution selected using jModelTest = GTR, Rates among sites = G+I, No. of discrete Gamma Categories = 6, Gaps Data Treatment = Complete deletion, Select Codon Position = 1^st^+2^nd^+3^rd^+Noncoding Sites, ML Heuristic Method = Subtree-Pruning-Regrafting – Extensive (SPR level 5), Initial Tree for ML = Make initial tree automatically (Default – NJ/BioNJ). The BI analyses were run with four parallel Markov chains with the following parameters: MCMC (Markov Chain Monte Carlo) generations = 20000000, sampling frequency = 1000, print frequency = 1000, number of runs = 2, number of chains = 4, MCMC burnin = 2500, sumt burnin = 2500, sump burnin = 2500, Models of sequence evolution selected using jModelTest = GTR, Rates among sites = G+I, Select Codon Position = 1^st^, 2^nd^, and 3^rd^. TRACER v. 1.6 (Rambaut and Drummond 2003–2009) was used to analyze the parameters and the effective sample size (ESS) of the MCMC to ensure the runs converged. FigTree v. 1.4.3 was used to visualize the topology of the tree with the posterior probability values (PP) at nodes. The ABGD species delimitation method uses recursive partitioning with a range of prior intraspecific divergence and relative gap widths, estimating the threshold between intra- and interspecific genetic variation, generating species-level groupings (Ortiz and Francke 2016). ABGD analyses were conducted using both uncorrected and K2P distance matrices with default options: Pmin = 0.001, Pmax = 0.1, Steps = 10, Relative gap width (X) = 1, Nb bins = 20. The GMYC species delimitation method applies single (Pons et al. 2006) or multiple (Monaghan et al. 2009) time thresholds to delimit species in a Maximum Likelihood context, using ultrametric trees (Ortiz and Francke 2016). Phylogenetic analyses were run in BEAST v. 2.6.0 (Drummond et al. 2012) using a coalescent (constant population) tree prior. Independent lognormal relaxed clock was applied to each partition, for analyses of 20×10^6^ generations were run. Convergence was assessed with TRACER v. 1.6 (Rambaut and Drummond 2014). TREEANNOTATOR v. 2.6.0 (BEAST package) was used to build maximum clade credibility trees, after discarding the first 25% of generations by burn-in. Following gene tree inference, GMYC was implemented in the web interface for single and multiple threshold GMYC (https://species.h-its.org/gmyc/) the backend of this web server runs the original R implementation of the GMYC model authored by Fujisawa and Barraclough (2013). A single threshold was used for the concatenated matrix. The PTP species delimitation method (Zhang et al. 2013) is similar to GMYC, but uses substitution calibrated (not ultrametric) trees to avoid the potential flaws in constructing time calibrated phylogenies (Zhang et al. 2013, Ortiz and Francke 2016). We employed the Bayesian variant of the method (bPTP) on the online version (https://species.h-its.org/ptp/). It was run on the Bayesian gene trees with default options: rooted tree, MCMC generations = 100000, Thinning = 100, Burnin = 0.1, Seed = 123. Haplotypes network for CO1 was constructed to visualize the mutations among haplotypes of species using the TCS algorithm (Clement et al. 2000) in PopArt v. 1.7 (Leigh and Bryant 2015).

### Geometric and linear morphometry and sexual dimorphism

For the morphometric studies, tibiae of adult males in retrolateral views of *L. tenochtitlan* sp. nov. (*N* = 12) and *L. misteca* (*N* = 9) were analyzed using Make Fan 8 v. 1.0 software (Sheets and Zelditch 2014), performing brand and semi-brand protocols. Using TPsUtil v. 1.76 software (Rohlf 2015) the file was formatted (.tps) to perform the digitalization of the landmarks and semi-landmarks of the contours in the tpsDig v. 2.31 software (Rohlf 2015). In the CordGen8 v. 1.0 software (Sheets and Zelditch 2014), a “Procrustes” alignment was made for the brands and with the Semi Land option included in the CordGen8 v. 1.0 software (Sheets and Zelditch 2014). Posteriorly, the alignment of the semi-landmarks was carried out. To analyze the formation of groups in relation to the tibia shape, an analysis of canonical variables (CVA) was performed with the CVA Gen 8 v. 1.0 software. To analyze sexual dimorphism and variation in the new species, a T-test was performed to evaluate if the females and males have significant statistical differences in: 1) leg I length, 2) carapace length, and 3) carapace width. Also, leg I length was used to test if differences exist between the new species and *Loxosceles misteca* Gertsch, 1958; species that appears to be closely related to *L. tenochtitlan* sp. nov. morphologically. Forty specimens of *Loxosceles tenochtitlan* sp. nov. (24 females and 16 males) and 22 specimens of *L. misteca* (11 females and 11 males) were measured (Table 5). The statistical analysis was carried out and graphics were made with R studio v.1.1.463 software.

### Ecological niche modeling (ENM)

For georeferencing and corroboration of localities, two programs were used: GeoLocate online version (http://www.museum.tulane.edu/geolocate/) and Google Earth v. 7.1.5.1557. The geographic coordinates were transformed from NAD83 to WGS84 online on INEGI: Transformation of coordinates TRANINV (INEGI 2019). Geographical coordinates are given in degrees. ENM data were generated using Maxent v. 3.3 (Maximum Entropy Algorithm) (Phillips et al. 2004) which estimates the probability of the presence of a lineage by looking for the distribution of maximum entropy (as uniform as possible) based on both quantitative and qualitative environmental variables. The AUC (Area Under the Curve) variable measures the ability of models to discriminate true and false positives for ENM using the following scale: excellent (AUC> 0.90), good (0.80> AUC <0.90), acceptable (0.70> AUC <0.80) (Phillips et al. 2006; Phillips and Dudík 2008; Illoldi-Rangel and Escalante 2008). ENM was conducted using 19 climatic variables: 17 from WorldClim v.1.0. (http://www.worldclim.org/) (BIO1-BIO19) (Fick and Hijmans 2017) and two from CONABIO (http://www.conabio.gob.mx/informacion/gis/) (CON01: vegetation type, and CON02: level curves for the Mexican Republic) (CONABIO, 2015). The climatic variables were previously processed in QGIS v. 2.18 “Las Palmas” to be read in MaxEnt. The ENM prediction and distribution maps were made using QGIS. Maps were edited using Adobe Photoshop CS6.

## Taxonomy

### Family Sicariidae Keyserling, 1880

#### Genus Loxosceles

Taxon classificationAnimaliaAraneaeSicariidae

Heineken & Lowe, 1832

95A31C6A-18CC-5309-853A-B3BA3A3DE815

##### Type species.

*Loxosceles rufescens* (Dufour, 1820).

#### Loxosceles tenochtitlan

Taxon classificationAnimaliaAraneaeSicariidae

Valdez-Mondragón & Navarro-Rodríguez sp. nov.

D519C07A-29E9-51DB-9ED8-93FF9F079515

http://zoobank.org/C87C7B99-E4A7-41CC-8F79-4229A05BBDB9

Figs 1–6 , 7–9 , 13 , 14–17 , 19–22 , 23–28 , 32–34 , 35–37 , 48–55 , 56–61

##### Type material.

MEXICO: *Mexico City*: male holotype (LATLAX-T001) from Street Cruz Verde No. 132 (lat 19.2921, lon -99.174203; 2281 m), Tlalpan, 10-XII-2017, M. Sánchez-Vílchis leg. (inside house). Paratypes: 1 female (LATLAX-T002), 2 males (LATLAX-T003), 4 females (LATLAX-0004), same data as holotype.

##### Other material examined.

MEXICO: *Mexico City*: 5 females, 1 immature (LATLAX-Ara 0539), same data as holotype. 3 males, 8 immatures (LATLAX-Ara 0540), same data as holotype. 1 male (LATLAX-Ara 0542), 19-I-2019, A. Valdez leg., same locality as holotype. 1 female, 1 immature (LATLAX-Ara0156) from Street Tepocatl #61, Pedregal de Santo Domingo (19.330101, -99.147210; 2256 m), Coyoacán, 02-VII-2017, R. Cansino López leg. 1 male (LATLAX-Ara1087) from Pedregal de Santo Domingo, (19.328704, -99.164989, 2273 m) Coyoacán, 21-VII-2017 R. Cansiano Lopéz leg. 1 male, 1 female, 1 immature (LATLAX-Ara 0193) Los Reyes Copilco, Fracc. Areada Dpto. 102-A (19.336984, -99.182979, 2272 m), Coyoacán, IX-2017, D. Guerrero leg. 1 female, 1 immature (LATLAX-Ara196) Los Reyes Copilco, Fracc. Areada Dpto. 102-A (19.336984, -99.182979, 2272 m), Coyoacán, XII-2017, D. Guerrero leg. 1 immature (LATLAX-Ara 0482) from Street Toriello Guerra, Cuitlahuac S/N (19.297228, -99.174510, 2269 m) Tlalpan, II-2018, D. Barrales leg. 1 male 1 immature (LATLAX-Ara 0487) from Street Toriello Guerra, Cuitlahuac S/N (19.297228, -99.174510, 2269 m) Tlalpan, II-2018, D. Barrales leg. 1 female (LATLAX-Ara 0507) from Street Tepocatl #61, Pedregal de Santo Domingo (19.330101, -99.147210; 2256 m), Coyoacán 09-VIII-2018, R. Cansino Lopéz leg. *Estado de Mexico*: 1 female (LATLAX-Ara 0529) from Street Juaréz #23, San Mateo Ixtacalco (19.702460, -19.187150, 2355 m), Municipality Cuautitlán Izcalli 05-III-2019, M. Cortez. *Tlaxcala*: 1 male, 3 females, 15 immatures (LATLAX-Ara0132) from Street Reforma #5, Santiago Tlacochcalco (19.26939, -98.22303, 2245 m), Municipality of Tepeyanco, 06-VI-2017, M. Cortez, A. Juárez, J. Valerdi Cols. 1 female (LATLAX-Ara0188) from the Trinidad Tenexyecac (19.335588, -98.315688, 2241 m), Municipality of Ixtacuixtla of Mariano Matamoros, 02-III-2018, E. Briones leg. 2 males, 3 females, 10 immatures (LATLAX-Ara0500) from North Street Juárez #214, Huamantla downtown (19.3168, -97.92245, 2511 m), Municipality Huamantla, 15-V-2018, A. Valdez, I. Navarro, P. Solís, A. Cabrera, D. Montiel. Cols. 6 males, 5 females, 46 immatures (LATLAX-Ara0501) from Street North Juárez #214, Huamantla downtown (19.3168, -97.92245, 2511 m), Municipality Huamantla, 08-VI-2018, A. Valdez, I. Navarro, P. Solís, A. Cabrera, D. Montiel. Cols. 6 male, 2 females, 46 immatures (LATLAX-Ara0502) from Santiago Tlacochcalco (19.26939, -98.22303, 2245 m), Municipality of Tepeyanco, 25-IV-2018, P. Solís, I. Navarro A. Juárez, J. Valerdi Cols.

**Figures 1–6.**
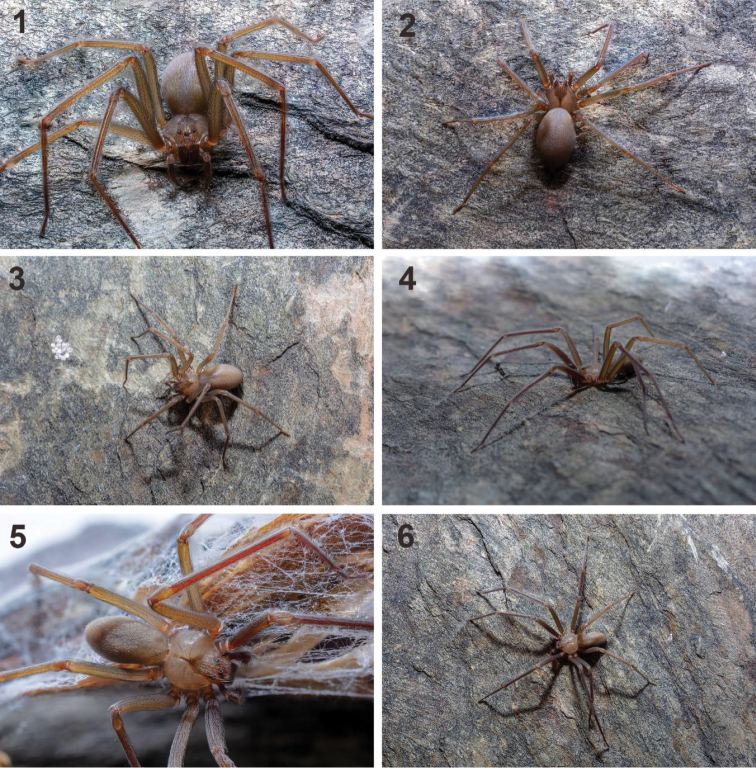
Live specimens of *Loxosceles tenochtitlan* sp. nov. from Street Juárez Norte #214, Huamantla, Municipality Huamantla, Tlaxcala, Mexico **1–3** females **4–6** males. Photographs by Jared Lacayo-Ramírez (2019).

**Figures 7–13.**
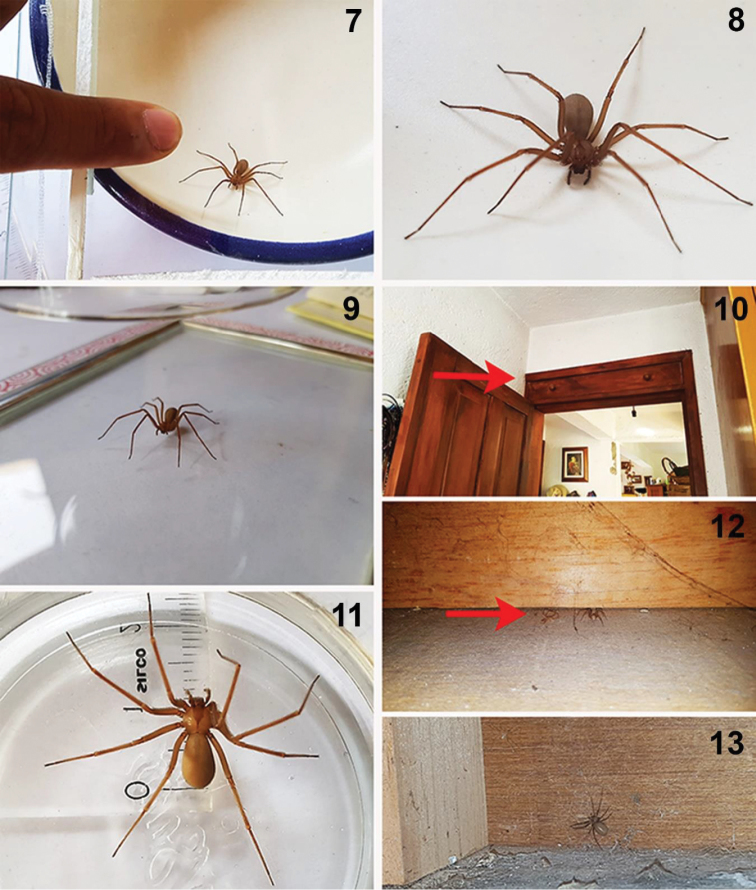
Live female specimens of the *Loxosceles tenochtitlan* sp. nov. from Street Cruz Verde #132, Tlalpan, Mexico City, Mexico (type locality). Red arrows indicate specific places where the specimens were collected inside the house. Photographs by Martín Sánchez Vílchis (2019).

**Figures 14–18.**
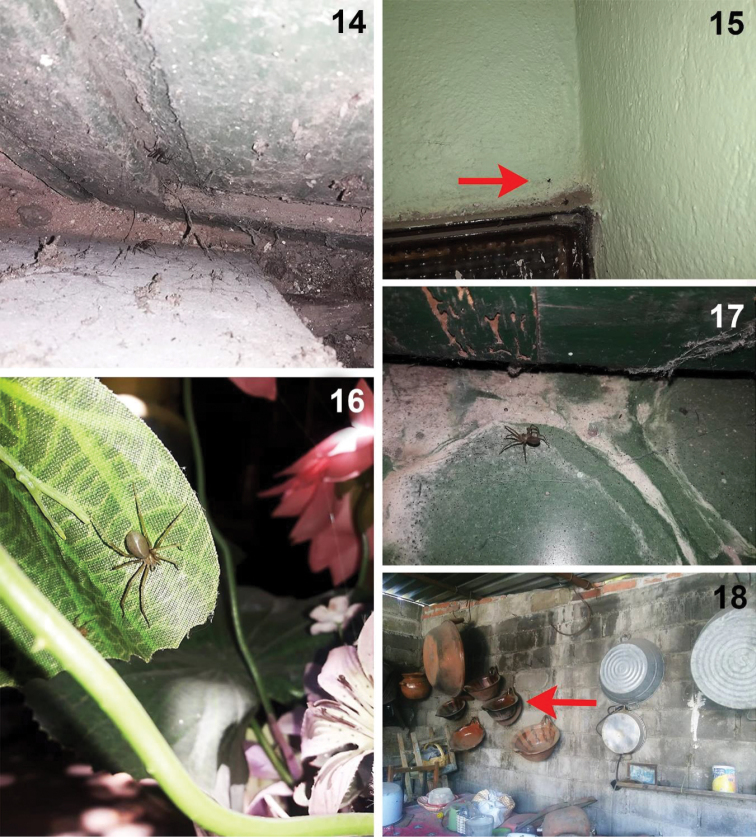
Live specimens and urban microhabitat of *Loxosceles tenochtitlan* sp. nov. from Tlaxcala **14–17** specimens from Street Juárez Norte #214, Huamantla downtown, Municipality, Huamantla, Tlaxcala, Mexico **18** microhabitat where some specimens were collected from Street Reforma #5, Santiago Tlacochcalco, Municipality Tepeyanco, Tlaxcala, Mexico. Red arrows indicate the specific places where specimens were collected. Photographs **14–17** by José A. Castilla-Vázquez (2018–2019). Photograph **18** by Alma R. Juárez-Sánchez (2018).

##### Etymology.

The species is a noun in apposition dedicated to Tenochtitlán (Nahuatl language) city, a large Mexica city-state in what is now Mexico City where the type locality is located. Tenochtitlán was built on an island in what was then Lake Texcoco in the Valley of Mexico, being the capital of the expanding Aztec Empire in the 15^th^ century.

##### Diagnosis.

The male of *Loxosceles tenochtitlan* sp. nov. morphologically resembles those of *Loxosceles misteca* Gertsch, 1958 (Figs 29–31, 38–47) from Guerrero; however, in the new species, the curvature of the basal-ventral part of the tibia of the male palp is less pronounced than in *L. misteca*¸ where it is prominent (Figs 23, 25, 42, 44, 48–55). Both species have a spatula-shaped embolus; in the new species, the embolus is slightly wider than that of *L. misteca* (Figs 23, 25, 26, 42, 44, 45, 48–55, 62–65). In dorsal view, the embolus basally is wider in *L. tenochtitlan* sp. nov. than in *L. misteca* (Figs 26, 45). Leg I length of males of *L. tenochtitlan* sp. nov. is shorter than legs I of *L. misteca* (Fig. 81). The seminal receptacles of females of *L. tenochtitlan* sp. nov. and *L. misteca* are similar, however in the new species the distance between the base of the receptacles is larger than in *L. misteca* (Figs 56–61, 66–69), also, the genitalia of *L. tenochtitlan* sp. nov. has small accessory lobes receptacles on each side (Figs 56–61), which are absent on *L. misteca* (Figs 66–69).

**Figures 19–22.**
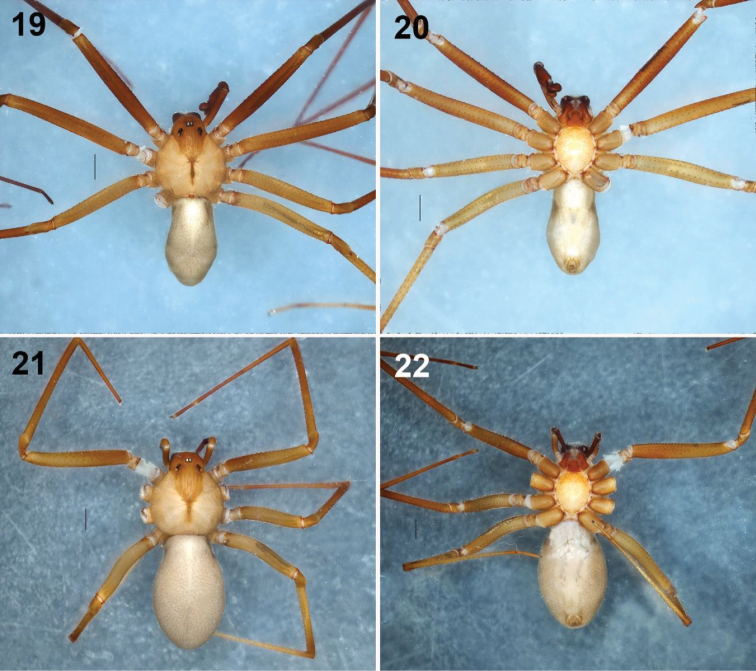
*Loxosceles tenochtitlan* sp. nov **19–20** habitus of male holotype, dorsal and ventral views, respectively **21–22** habitus of female paratype, dorsal and ventral views, respectively. Scale bars: 1 mm.

##### Description.

**Male (holotype; LATLAX-T001)**: Specimen collected manually, preserved and observed in 80% ethanol. *Measurements*: Total length (prosoma + opisthosoma) 6.70. Carapace 3.20 long, 2.90 wide. Clypeus length 0.45. Diameter of AME 0.13, PME 0.17, PLE 0.20; AME-PME 0.20 Labium: length 0.79, width 0.58. Sternum: length 1.80, width 1.62. Leg lengths: I (total 18.55): femur 5.00 / patella 1.10 / tibia 5.90 / metatarsus 5.35 / tarsus 1.20; II (20.98): 5.60 / 1.12 / 6.75 / 6.20 / 1.31; III (15.67): 4.40 / 1.10 / 4.45 / 4.60 / 1.12; IV (16.99): 4.75 / 1.02 / 4.92 / 5.10 / 1.20. Leg formula: 2-1-4-3.

***Prosoma***: Carapace orange, longer than wide, piriform, with small and numerous setae laterally, with defined pale brown violin-shaped pattern dorsally, darker toward ocular region, carapace without spots (Fig. 19). Fovea brown (Fig. 19). Six eyes in three groups, clypeus reddish orange. Sternum pale orange, longer than wide (Fig. 20). Labium reddish, longer than wide, trapezoidal, fused to the sternum (Fig. 20). Endites pale orange basally, reddish distally and white apically, longer than wide, rounded basally (Fig. 20).

***Legs***: Coxae pale orange (Fig. 20). Trochanters pale orange. Femora pale orange, reddish orange on femora I (Figs 19, 20). Patellae dark orange. Tibiae, metatarsi and tarsi reddish orange.

***Chelicerae***: Fused basally, chelated chelicerae laminae, reddish orange, stridulatory lines laterally. Fangs reddish orange, paler distally, with long and thin setae.

***Opisthosoma***: Pale yellow, darker posteriorly, oval, longer than both width and height (Figs 19, 20). Region of gonopore pale yellow (Fig. 20), surrounded by small setae. Colulus long, pale orange, conical. Spinnerets pale orange, anterior lateral spinnerets cylindrical, longest, posterior median spinnerets shortest, with long setae; posterior lateral spinnerets cylindrical, slightly curved and with some long setae. Tracheal opening near posterior margin of opisthosoma.

***Palps***: Trochanters orange, femora reddish brown, long and thin, patellae reddish brown; tibiae reddish orange, darker, oval, curved ventrally, almost straight dorsally, wider distally than ventrally (Figs 23–25). Tarsus oval, reddish brown, bulb oval, with short, wide and slightly curved embolus (Figs 26–28, 32–34, 35, 36). Canal along embolus (Figs 32–34, 37).

**Figures 23–28.**
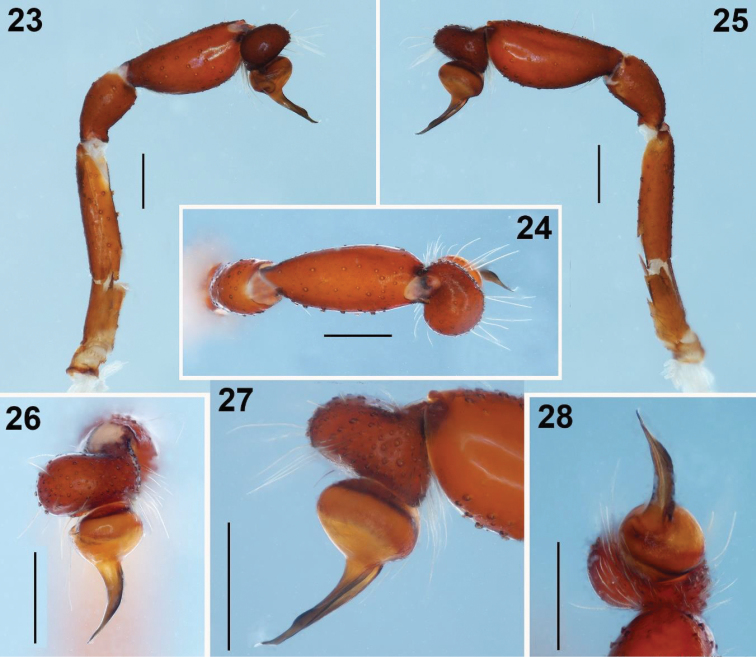
*Loxosceles tenochtitlan* sp. nov. Male Holotype **23–25** left palp, prolateral, dorsal and retrolateral views respectively **26–28** detail of the bulb and embolus, dorsal, retrolateral and apical views, respectively. Scale bars: 0.5 mm (**23–25**), 0.2 mm (**26–28**).

**Female (paratype; LATLAX-T002)**: Specimen collected manually, preserved and observed in 80% ethanol. Measurements: Total length 10.40. Carapace 3.75 long, 3.25 wide. Clypeus length 0.55. Diameter of AME 0.16, PME 0.20, PLE 0.20; AME-PME 0.23 Labium: length 0.80, width 0.75. Sternum: length 2.05, width 1.75. Leg lengths: I (total 18.73): femur 5.10 / patella 1.20 / tibia 5.68 / metatarsus 5.50 / tarsus 1.25; II (19.79): 5.50 / 1.24 / 6.10 / 5.60 / 1.35; III (15.83): 4.50 / 1.25 / 4.50 / 4.50 / 1.08; IV (18.09): 5.10 / 1.20 / 5.18 / 5.37 / 1.24. Leg formula: 2-1-4-3.

Differs from the male as follows. *Prosoma*: Carapace paler orange, with darker brown violin-shaped pattern; ocular region dark brown (Fig. 21). Clypeus dark reddish orange. Sternum dark orange (Fig. 22). Labium and endites more reddish orange, endites flat basally (Fig. 22). *Legs*: Coxae dark orange (Figs 21, 22). Trochanters dark orange (Fig. 22). All femora pale orange (Figs 21, 22). Patellae dark orange. Tibiae, metatarsi, and tarsi pale reddish orange (Figs 21, 22).

***Chelicerae***: Wider than in the male. Slightly dark reddish brown, with stridulatory lines laterally. Fangs dark reddish orange.

***Opisthosoma***: Opisthosoma pale yellow (Figs 21, 22). Spinnerets dark orange.

***Palps***: Trochanters pale orange, femora pale brown, paler ventrally; patellae pale brown, tibiae and tarsi reddish surrounded with several long and sparse setae. Tibiae cylindrical, tarsi conical.

***Genital area***: Seminal receptacles asymmetric, S-shaped, curved basally and apically with rounded lobes (Fig. 56). Base of seminal receptacles wide and slightly sclerotized, round internally (Fig. 56). See variation section for more details (Figs 57–61).

##### Variation.

MALES. *Mexico City*: Males from Coyoacán are light brown, legs slightly darker than the carapace, males from Tlalpan are light brown, legs slightly darker than the carapace. *Tlaxcala*: Males from Santiago Tlacochcalco Municipality of Tepeyanco are light brown, legs slightly darker than the carapace and light brown, legs slightly darker than the carapace. Males from Huamantla are dark brown, legs slightly darker than the carapace. *Mexico City*: Coyoacán (*N* = 3): Tibia I 5.9–6.5 (x̄ = 6.1); carapace length (CL) 2.6–3.1 (x̄ = 2.9); carapace width (CW) 2.4–2.7 (x̄ = 2.5). Tlalpan (*N* = 3): Tibia I 6.0–7.6 (x̄ = 5.8); carapace length (CL) 2.2–3.2 (x̄ = 2.8); carapace width (CW) 2.5–2.7 (x̄ = 2.6). *Tlaxcala*: Santiago Tlacochcalco Municipality of Tepeyanco (*N* = 7): Tibia I 3.8–6.6 (x̄ = 5.0); carapace length (CL) 2.5–4.2 (x̄ = 3.1); carapace width (CW) 2.2–3.2 (x̄ = 2.7). Huamantla (*N* = 3): Tibia I 5.0–6.5 (x̄ = 5.8); carapace length (CL) 3.2–3.3 (x̄ = 3.2); carapace width (CW) 2.7–2.9 (x̄ = 2.8). FEMALES. *Mexico City*: Females from Coyoacán are dark brown, legs the same color as the carapace. Females from Tlalpan are dark brown, legs the same color as the carapace. *Estado de Mexico*: Female from San Mateo Ixtacalco, Municipality Cuautitlán Izcalli is dark brown, legs slightly darker than the carapace. *Tlaxcala*: Females from Santiago Tlacochcalco, Municipality of Tepeyanco are light brown, legs slightly darker than the carapace. Females from Huamantla are dark brown, legs the same color as the carapace and light brown, legs the same color as the carapace and light brown. A female from the Trinidad Tenexyecac, Municipality of Ixtacuixtla is light brown, legs the same color as the carapace. *Mexico City*: Coyoacán (*N* = 3): Tibia I 5.8–7.1 (x̄ = 6.7); carapace length (CL) 3.9–4.2 (x̄ = 4.1); carapace width (CW) 3.2–4.0 (x̄ = 3.7). Tlalpan (*N* = 6): Tibia I 4.6–6.3 (x̄ = 5.2); carapace length (CL) 1.7–4.0 (x̄ = 3.2); carapace width (CW) 1.8–3.3 (x̄ = 2.6). *Estado de Mexico*: San Mateo Ixtacalco, Municipality Cuautitlán Izcalli (*N* = 1) Tibia I 3.6; carapace length (CL) 2.5; carapace width (CW) 2.5. *Tlaxcala*: Santiago Tlacochcalco Municipality of Tepeyanco (*N* = 2): Tibia I 4.5, 5.3; carapace length (CL) 3.2, 3.3; carapace width (CW) 2.5, 2.9. Huamantla (*N* = 11): Tibia I 4.1–6.7 (x̄ = 5.1); carapace length (CL) 1.7–4.0 (x̄ = 3.3); carapace width (CW) 1.8–3.5 (x̄ = 2.7). Trinidad Tenexyecac, Municipality of Ixtacuixtla (*N* = 1): Tibia I 5.4; carapace length (CL) 3.3; carapace width (CW) 2.5.

There is little variation in the shape of the male palps, even those of specimens from different populations (Figs 48–55). The shape of the embolus varies little; the specimens from Tlaxcala have the embolus slightly more curved than the specimens from Mexico City (Figs 52–55). Also, the specimens from Tlaxcala have a slightly thinner palpal tibia than specimens from Mexico City (Figs 48–51). The seminal receptacles of females are asymmetrical, and although all they are all S-shaped with rounded or oval lobes apically, they are highly variable (Figs 56–61). The small accessory lobes of the receptacles on each side vary in width among specimens (Figs 56–61). The internal part of the bases of the seminal receptacles is round, wide and slightly sclerotized in all specimens, with the distance between them equal to their height (Figs 56–61).

##### Natural history.

The specimens of *L. tenochtitlan* sp. nov. (Figs 1–9, 11–17) were collected in urban areas in houses and buildings (Figs 10, 18). The specimens from Mexico City were collected in houses, on doors, storage boxes, drawers, under chairs and tables (Figs 7–13). The specimens from Tlaxcala were collected in houses behind doors, behind decorative items on the wall, under beds, under chairs and tables, among wooden boards for construction, under wardrobes, and between ornamental artificial plants, and under stored items (Figs 14–18). Even the first record from Tlaxcala (Trinidad Tenexyecac) was a female specimen collected among construction debris close to a football/soccer field. Some specimens from Huamantla, Tlaxcala were collected inside an abandoned house, mainly under stored items, behind doors and under wardrobes; other specimens were collected outside of a house in spaces and cracks in a wall (Figs 14–17).

##### Distribution.

MEXICO: Mexico City, Tlaxcala, Estado de Mexico (Figs 82–84).

#### Loxosceles misteca

Taxon classificationAnimaliaAraneaeSicariidae

Gertsch, 1958

9E3CCCFE-7D24-5A30-9925-F22886A29CE2

Figs 29–31 , 38–41 , 42–47 , 62–69

##### Type material.

MEXICO: *Guerrero*: male holotype (examined) (AMNH_IZC00327631) from Taxco, Municipality Taxco de Alarcón, Guerrero, Mexico, Date? 1946, Collected in the fall, Leo Isaacs leg.

##### Material examined.

MEXICO: *Guerrero*: 1 male, 1 female (CNAN-AR008985) from Cueva del Diablo, Acuitlapan (18.60106, -99.54318, 1581 m) Municipality Taxco de Alarcón, 04-VI-2010, O. Francke, D. Barrales, J. Cruz, A. Valdez Cols. 2 males (LATLAX-Ara 0158) from Cueva del Jardín Botánico, Parque Nacional Grutas de Cacahuamilpa (18.67038, -99.51134, 1145 m) Municipality Pilcaya, 15-IX-2017, A. Valdez, P. Solís, I. Navarro, J. Valerdi Cols. 2 males (LATLAX-Ara 0161) from Grutas del General Pacheco (18.66562, -99.50943, 1086 m) Municipality Pilcaya, 19-IX-2017, A.Valdez, P. Solís, I. Navarro, J. Valerdi Cols. 6 females (LATLAX-Ara 0162) from Cueva Agustín Lorenzo, Mexcaltepec (18.431,-99.55013, 922 m) Municipality Taxco de Alarcón, 20-IX-2017, A. Valdez, P. Solís, I. Navarro, J. Valerdi Cols. 3 males, 5 females (LATLAX-Ara 0526) from Jardín Botánico, Parque Nacional Grutas de Cacahuamilpa (18.67038, -99.51134, 1145 m) Municipality Pilcaya, 15-X-2019, A. Valdez, P. Solís, I. Navarro, A. Juaréz, A. Cabrera Cols. *Morelos*. 1 male (CNAN-Ar009069) from Lomas de Cortés, Municipality Cuernavaca, 11-II-2013, P. Bernard leg. 1 male (CNAN-Ar009070) from Tlaltenango (18.946414, -99.24392, 1660 m) Municipality Cuernavaca, III-2013. R. Rosas leg. 1 male (CNAN-Ar009071) from Boulevard Cuahutémoc #33, Lomas de Cortés (18.951125, -99.22408, 1640) Municipality Cuernavaca, 24-II-2012.

**Diagnosis.***Loxosceles misteca* Gertsch, 1958 resembles *L. tenochtitlan* sp. nov. (Figs 23–28, 42–47); however, in *L. misteca*, the curvature of the basal-ventral part of the tibia of the male palp is more pronounced than in the new species (Figs 23, 25, 42, 44, 48–55, 62–65, 76–77). Both species have a spatula-shaped embolus; in *L. misteca*, the embolus is slightly thinner than that of the new species (Figs 23, 25, 42, 44, 48–55, 62–65, 76–77). Leg I length of males of *L. misteca* is longer than legs I of *L. tenochtitlan* sp. nov. (Fig. 81). The seminal receptacles of the females of *L. misteca* and *L. tenochtitlan* sp. nov. are similar, however in *L. misteca* the distance between the base of the receptacles is shorter than in the new species (Figs 56–61, 66–69), also, the genitalia of *L. misteca* does not have small accessory lobes receptacles on each side, which are present in *L. tenochtitlan* sp. nov. (Figs 56–61, 66–69).

**Figures 29–34.**
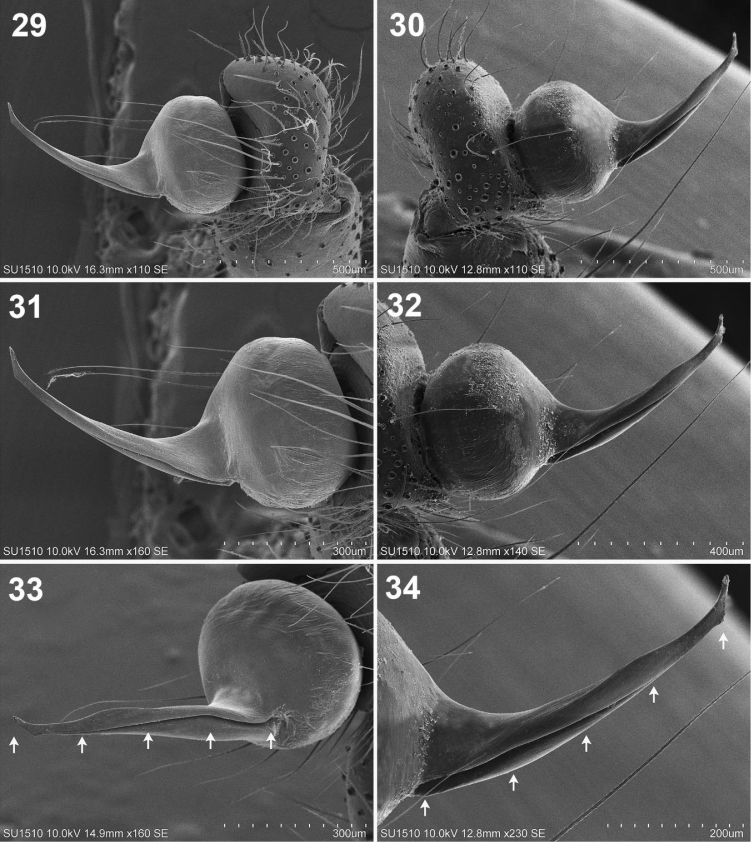
**29–31***Loxosceles misteca* Gertsch. Male **29** left palp, retrolateral view, detail of tarsus, bulb and embolus **30** detail of bulb and embolus, retrolateral view **31** detail of the embolus **32–34***Loxosceles tenochtitlan* sp. nov. Male paratype **32** right palp, retrolateral view, detail of tarsus, bulb and embolus **33** detail of bulb and embolus, retrolateral view **34** detail of the embolus. Arrows indicate the canal along the embolus.

**Figures 35–37.**
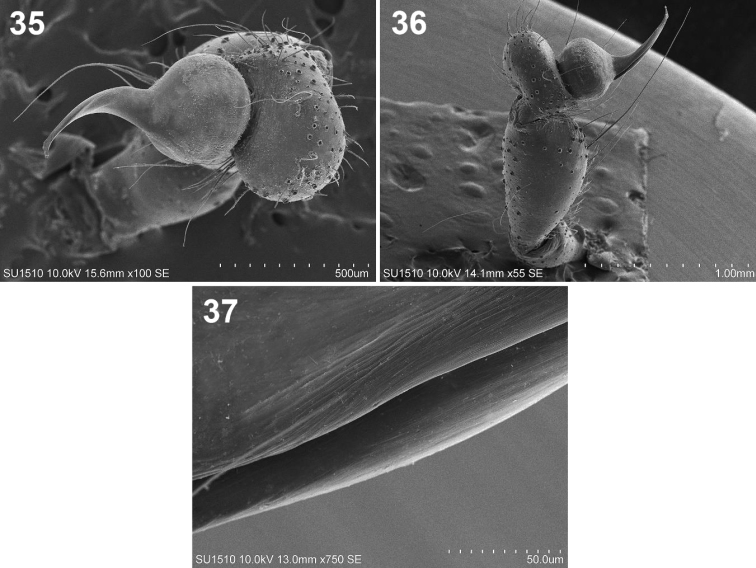
*Loxosceles tenochtitlan* sp. nov. Male paratype **35** right palp, tarsus, bulb and embolus, dorsal view **36** right palp, retrolateral view **37** detail of the canal along the embolus.

**Figures 38–41.**
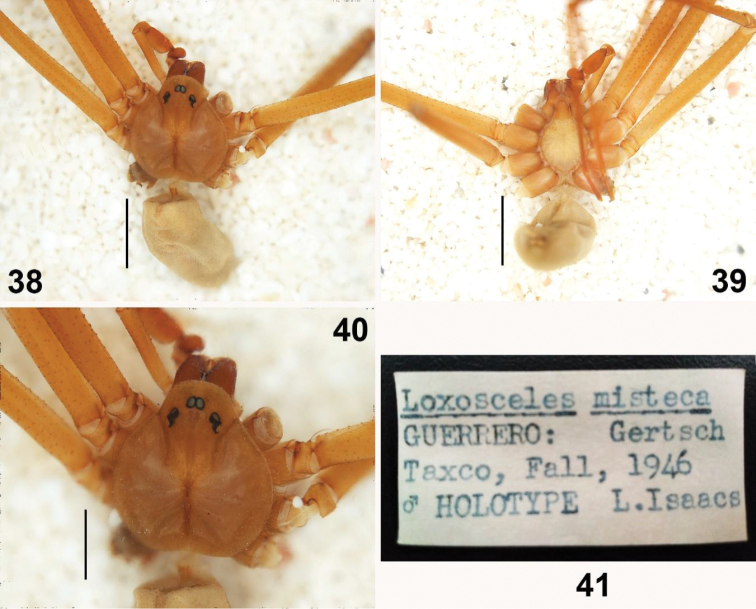
Male holotype (examined) of *Loxosceles misteca* Gertsch, 1958 (AMNH_IZC 00327631), from Taxco, Municipality Taxco de Alarcón, Guerrero, Mexico; Date? 1946, collected in the fall, Leo Isaacs leg. **38, 39** habitus of male holotype, dorsal and ventral views, respectively **40** carapace **41** label of the holotype. Scale bars: 1 mm (**38–40**).

**Figures 42–47.**
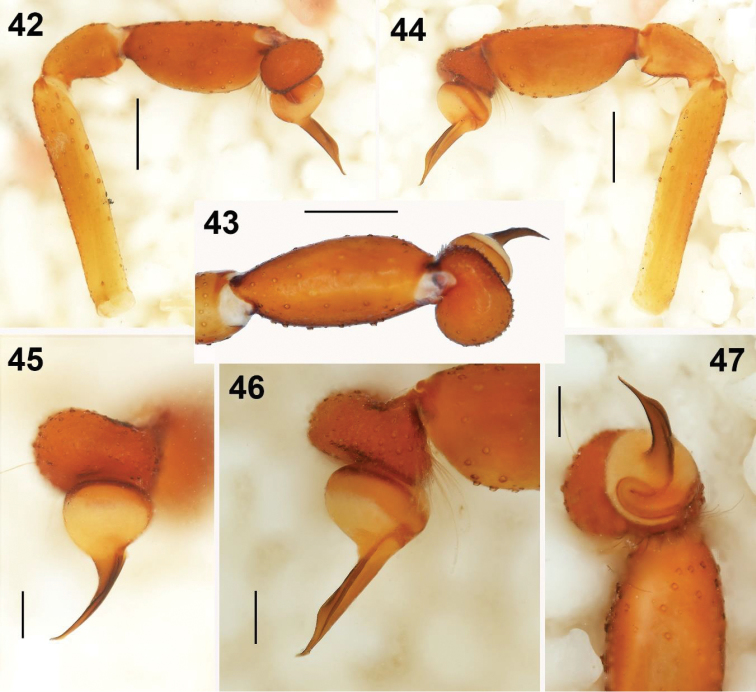
Male holotype (examined) of *Loxosceles misteca* Gertsch, 1958 (AMNH_IZC 00327631), from Taxco, Municipality Taxco de Alarcón, Guerrero, Mexico; 1946, collected in the fall, Leo Isaacs leg. **42–44** left palp, prolateral, dorsal and retrolateral views respectively **45–47** detail of the bulb and embolus, dorsal, retrolateral and apical views, respectively. Scale bars: 0.5 mm (**42–44**), 0.2 mm (**45–47**).

**Figures 48–55.**
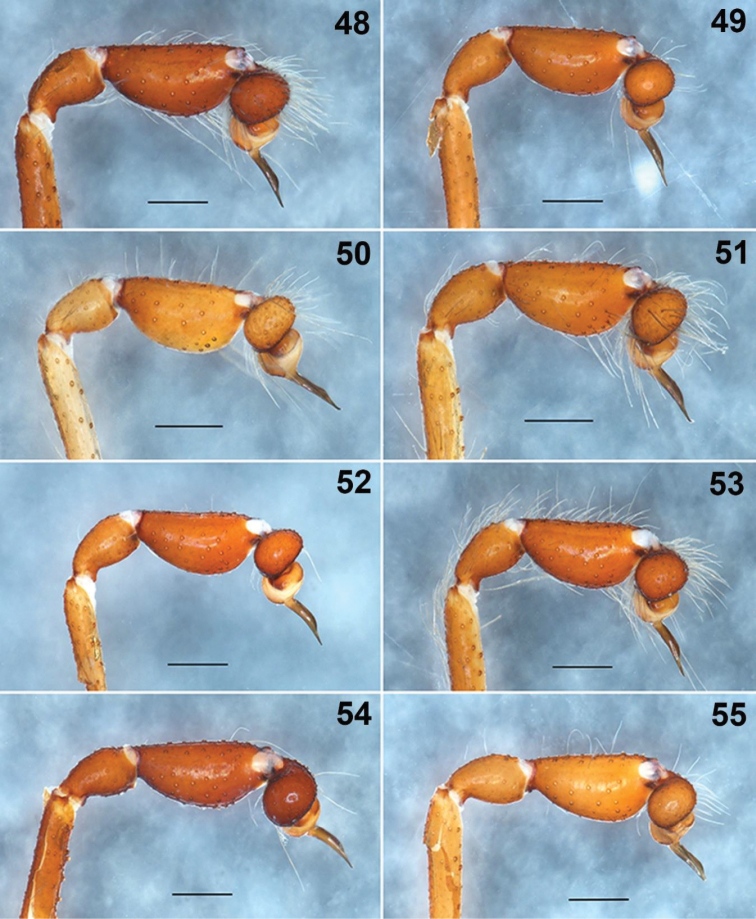
*Loxosceles tenochtitlan* sp. nov. Variation of the male palps, left palps, prolateral views **48** turiello Guerra, Street Cuitlahuac S/N, Tlalpan, Mexico City **49** Cruz Verde #132, Tlalpan, Mexico City (type locality) **50** Street Tepocatl #61, Pedregal de Santo Domingo, Coyoacán, Mexico City **51** Los Reyes Copilco, Frac. Areada Dpto. 102-A, Coyoacán, Mexico City **52, 53** Street Reforma #5, Santiago Tlacochcalco, Municipality of Tepeyanco, Tlaxcala **54, 55** Street Juárez Norte #214, Huamantla, Municipality Huamantla, Tlaxcala, Mexico. Scale bars: 0.5 mm.

**Figures 56–61.**
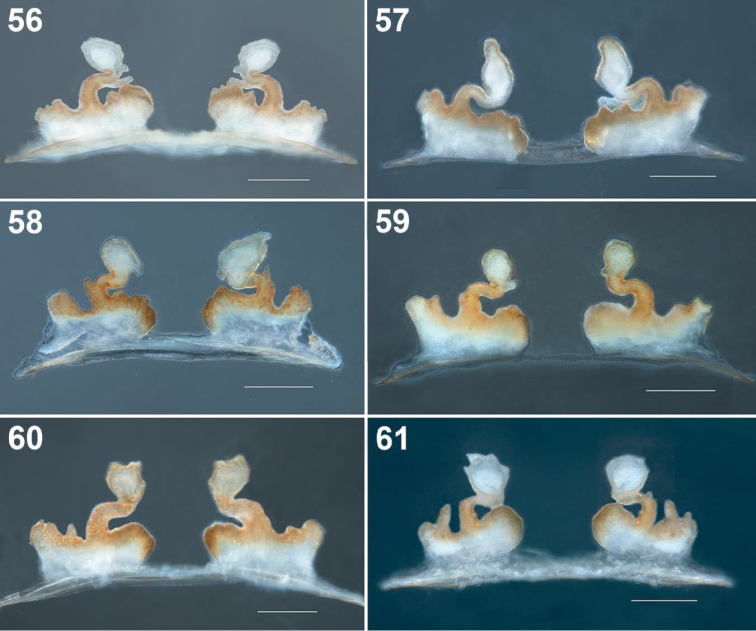
*Loxosceles tenochtitlan* sp. nov. Variation of the seminal receptacles of the females, dorsal views **56** Street Cruz Verde #132, Tlalpan, Mexico City (type locality) (female paratype) **57** Los Reyes Copilco, Fracc. Areada Dpto. 102-A, Coyoacán, Mexico City **58** Street Juárez #23, San Mateo Ixtacalco, Municipality Cuautitlán Izcalli, Estado de Mexico **59** Street Reforma #5, Santiago Tlacochcalco, Municipality of Tepeyanco, Tlaxcala **60, 61** Street Juárez Norte #214, Huamantla, Municipality of Huamantla, Tlaxcala, Mexico.

**Figures 62–69.**
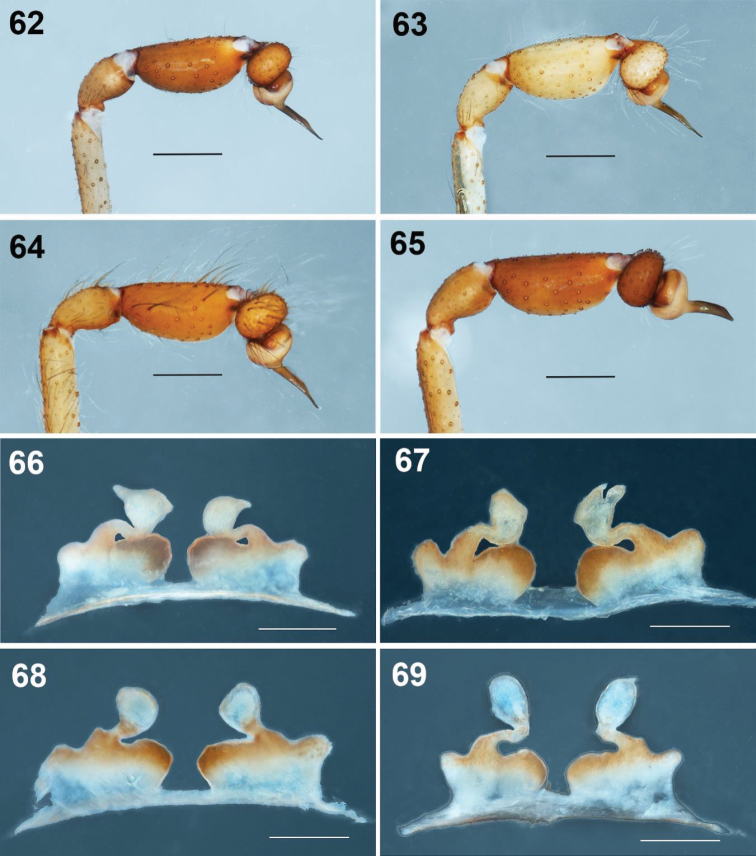
*Loxosceles misteca* Gertsch, 1958 **62–65** variation of the male palps, left palps, prolateral views **62** Grutas General Carlos Pacheco, Municipality Pilcaya, Guerrero **63** Cueva del Diablo Acuitlalpan, Municipality Taxco, Guerrero **64** boulevard Cuauhtémoc #99, Colonia Lomas de Cortes, Municipality Cuernavaca, Morelos **65** Grutas de Cacahuamilpa National Park, Municipality Pilcaya, Guerrero **66–69** variation of the seminal receptacles of the females, dorsal views **66, 67** Agustin Lorenzo Cave, Mexcaltepec, Municipality Taxco de Alarcón, Guerrero **68, 69** Botanical Garden Cave, Grutas de Cacahuamilpa National Park, Municipality Pilcaya, Guerrero.

### Molecular analyses and species delimitation

**Figure 70.**
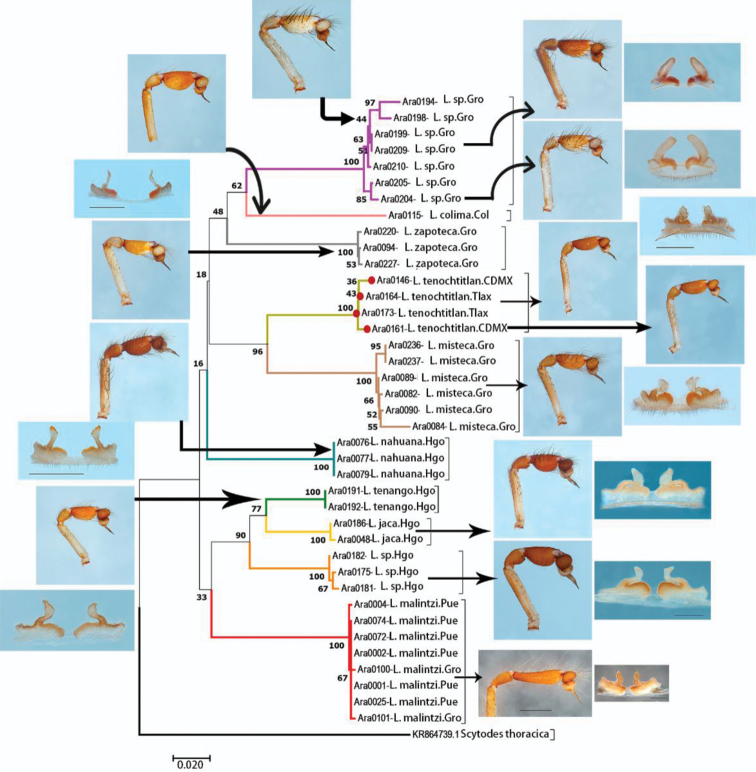
Neighbor-joining (NJ) tree constructed from COI data of ten species of *Loxosceles* from Mexico. Colors of branches indicate different species. Numbers on nodes are bootstrap support values. Red circles represent *Loxosceles tenochtitlan* sp. nov.

**Figure 71.**
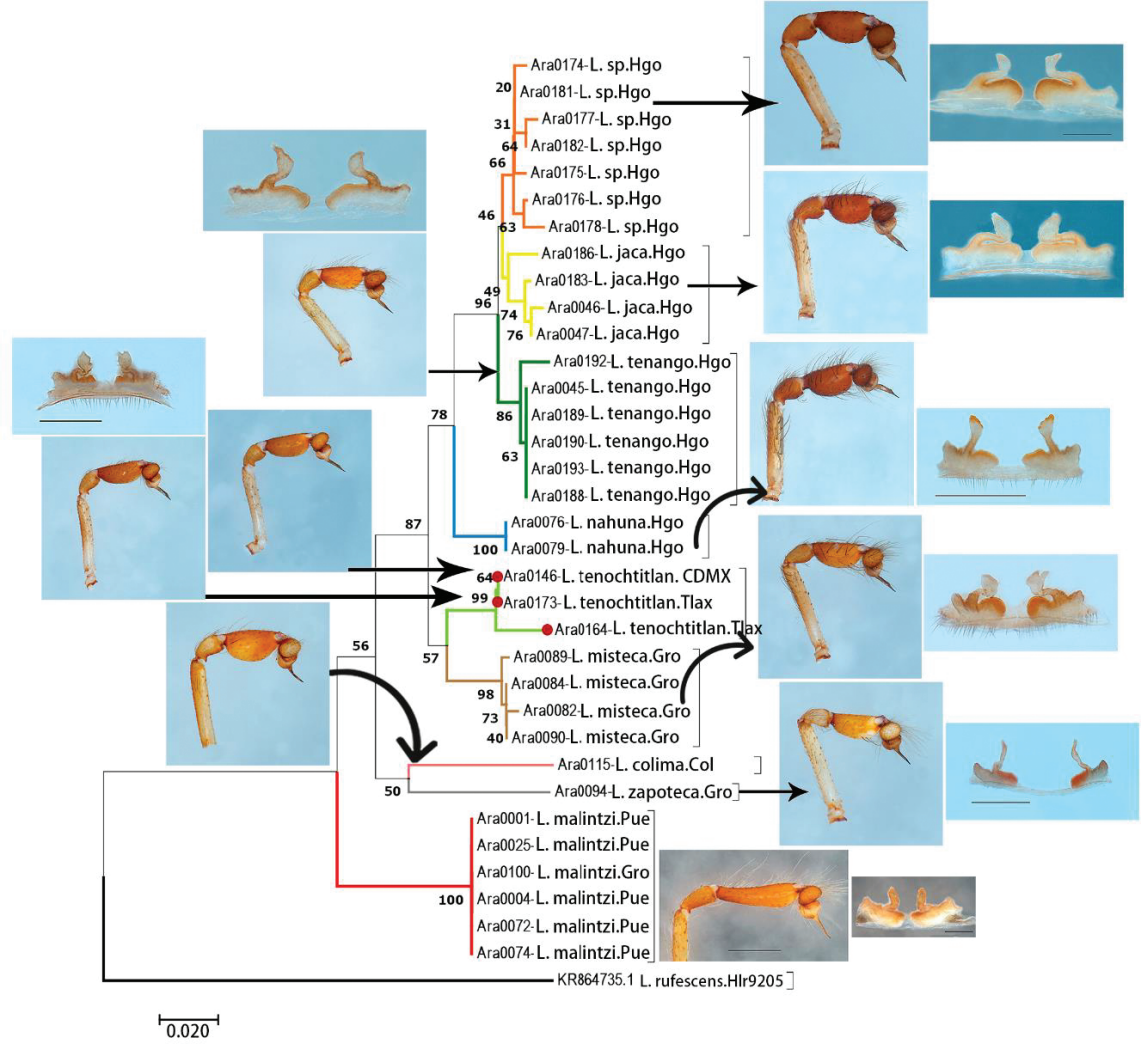
Neighbor-joining (NJ) tree of ITS2 data of nine species of *Loxosceles* from Mexico. Colors of branches indicate different species. Numbers at nodes represent bootstrap support values. Red circles represent *Loxosceles tenochtitlan* sp. nov.

**Figure 72.**
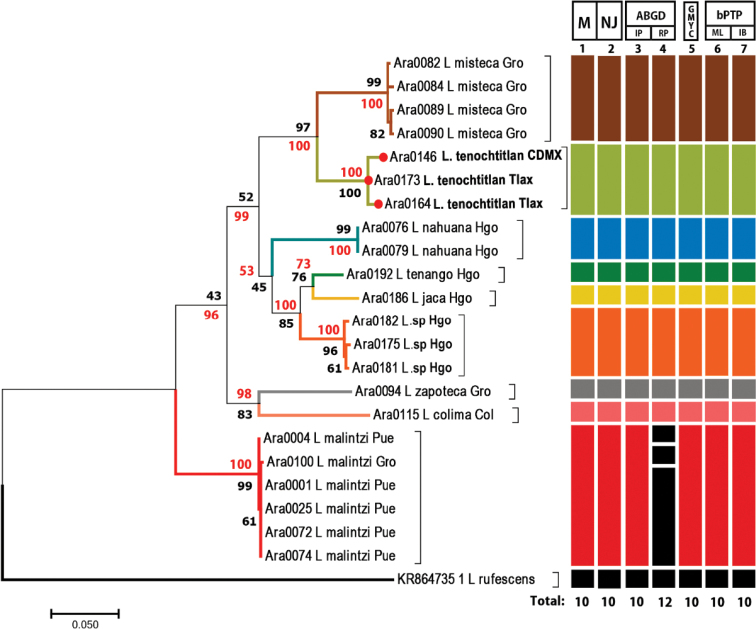
Maximum likelihood tree inferred from the concatenated matrix (CO1 + ITS2) of *Loxosceles* species from Mexico. Colors of branches and bars indicate different species. Numbers above bars at right represent the delimitation methods: 1: morphology (M). 2: neighbor-joining (NJ). 3: ABGD with initial partitions (IP). 4: ABGD with recursive partitions (RP). 5: GMYC. 6: bPTP with ML. 7: bPTP with IB. Numbers below bars represent species recovered for each delimitation method. Red numbers correspond to Bayesian posterior probabilities, and black numbers are bootstrap support values from the ML analysis.

**Figure 73.**
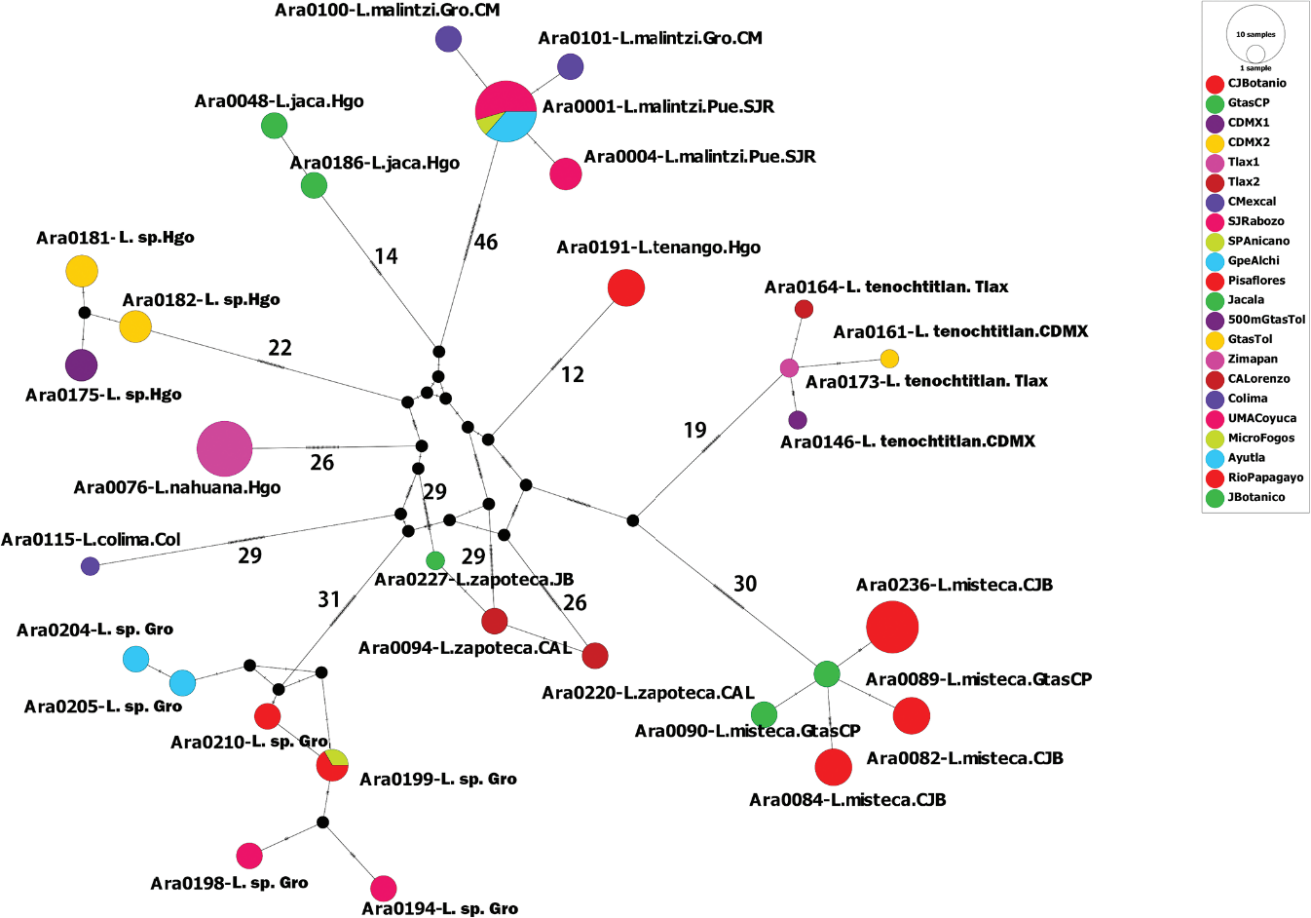
Haplotype network from the CO1 data obtained with TCS using PopArt. Each circle represents the haplotypes found in ten species of *Loxosceles* from Mexico. Numbers on branches indicate the number of mutations between haplotypes.

The analyzed matrices include 52 individuals of 11 species of *Loxosceles*, 39 individuals for the CO1 data set and 34 individuals for ITS2 (Table 1, Figs 70, 71). Specimens used in this study, GenBank accession numbers and localities of the specimens are listed in Table 1. Analyses of the concatenated matrix indicated that the four different methods used to delimit species with molecular data (CO1+ITS2) were consistent with morphology, recovering ten species (Fig. 72). Only the ABGD species delimitation method under recursive partitions (RP) recovered 12 species (Fig. 72). Even, *Loxosceles malintzi*, the last species described from Mexico by Valdez-Mondragón et al. (2018) by only morphological characters, was recovered with molecular data under the different species delimitation methods (Fig. 70–72). The average genetic *p*-distance among analyzed species was of 17% for CO1 and 7.6% for ITS2 (Figs 70, 71). Corrected *p*-distances from the CO1 data recovered ten species of *Loxosceles* (Fig. 70), whereas nine species were recovered with ITS2 (Fig. 71) both with high statistical support. Based on molecular evidence, *L. tenochtitlan* sp. nov. is closely related to *L. misteca* (Figs 70–72), the average *p*-distances between both species for CO1 was 13.8% (Table 3) and 4.2% for ITS2 (Table 4). The haplotype network analysis with CO1 data is concordant with the results of the different species delimitation analyses (Fig. 73). There were more than ten mutations between haplotypes of CO1 for all the species (Fig. 73). Regarding *L. tenochtitlan* sp. nov. and *L. misteca*, the haplotype network was concordant with the delimitation of both species, showed 49 mutations between haplotypes under CO1 (Fig. 73).

**Table 3.** Genetic *p*-distance matrix from the CO1 data between *Loxosceles tenochtitlan* sp. nov. and *Loxosceles misteca*. Average *p*-distance = 13.8%.

Species	1	2	3	4	5	6	7	8	9
1. Ara0082-*L. misteca* Gro									
2. Ara0089-*L. misteca* Gro	0.007								
3. Ara0090-*L. misteca* Gro	0.010	0.003							
4. Ara0084-*L. misteca* Gro	0.017	0.020	0.024						
5. Ara0236-*L. misteca* Gro	0.009	0.012	0.014	0.019					
6. Ara0237-*L. misteca* Gro	0.009	0.012	0.014	0.021	0.000				
7. Ara0146-*L. tenochtitlan* CDMX	0.150	0.153	0.153	0.166	0.155	0.157			
8. Ara0161-*L. tenochtitlan* CDMX	0.133	0.131	0.131	0.150	0.134	0.137	0.014		
9. Ara0173-*L. tenochtitlan* Tlax	0.122	0.126	0.126	0.136	0.124	0.126	0.019	0.006	
10. Ara0164-*L. tenochtitlan* Tlax	0.131	0.135	0.136	0.145	0.129	0.131	0.023	0.012	0.008

**Table 4.** Genetic *p*-distance matrix from the ITS2 data between *Loxosceles tenochtitlan* sp. nov. and *Loxosceles misteca*. Average *p*-distance = 4.2%.

**Species**	**1**	**2**	**3**	**4**	**5**	**6**
1. Ara0146-*L. tenochtitlan* CDMX						
2. Ara0173-*L. tenochtitlan* Tlax	0.000					
3. Ara0164-*L. tenochtitlan* Tlax	0.021	0.019				
4. Ara0082-*L. misteca* Gro	0.036	0.037	0.062			
5. Ara0084-*L. misteca* Gro	0.030	0.031	0.059	0.005		
6. Ara0090-*L. misteca* Gro	0.026	0.026	0.066	0.020	0.014	
7. Ara0089-*L. misteca* Gro	0.036	0.036	0.055	0.007	0.003	0.003

### Geometric and linear morphometry and sexual dimorphism

**Figures 74–77.**
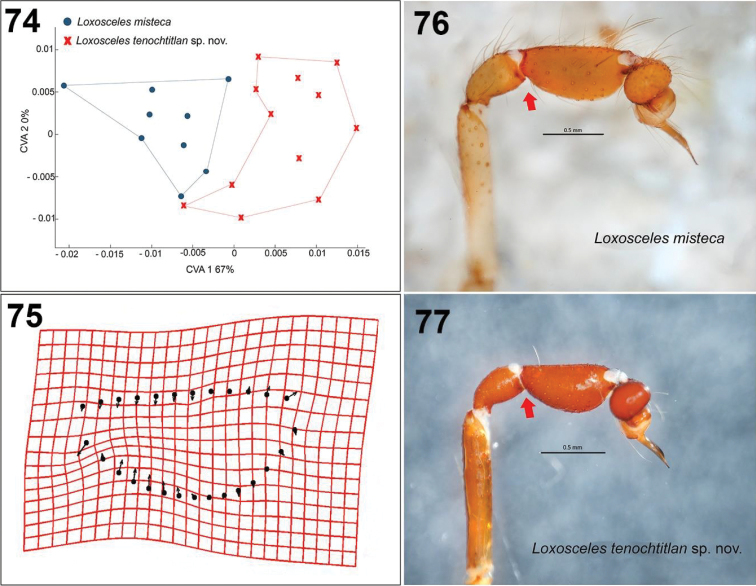
Geometric morphometry of the tibia shape on retrolateral view of the palps of males of *L. tenochtitlan* sp. nov. (*N* = 12) and *L. misteca* (*N* = 9) **74**CVA plot showing a significant difference (χ^2^ = 10.2555, df = 2, p = 0.00593003, λ = 0.5988) between both species in the tibiae shape **75** deformation grid, the vectors indicate the direction of change in the tibia with respect to the average shape of the 21 individuals analyzed of both species **76, 77** palps of the males of *L. misteca* and *L. tenochtitlan* sp. nov. respectively, retrolateral views (red arrows indicate the change in the shape of the tibiae of the species analyzed).

**Figures 78–81.**
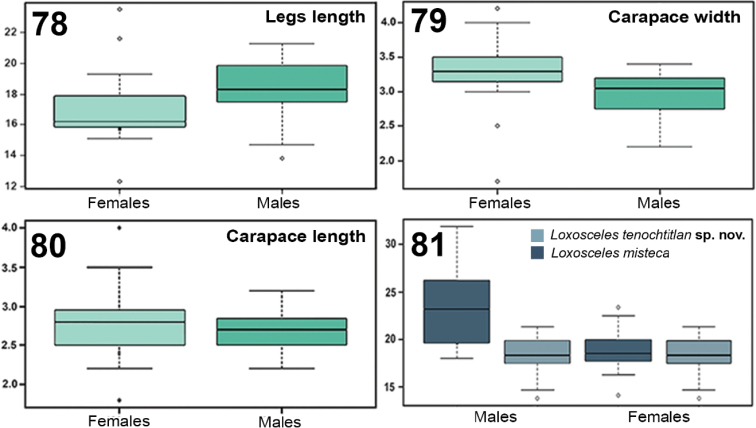
Sexual dimorphism of *Loxosceles tenochtitlan* sp. nov. (T test) **78** box plots showing the variation of leg length 1 between males and females (t = -1.3106, p = 0.1981, df = 37, α = 0.05) **79, 80** box plots showing variation of carapace length (**79**) and width (**80**) between males and females (length: t = 1.498, p = 0.142, df = *38*, α = 0.05; width: t = 0.6955, p = 0.4912, df = 36, α = 0.05) **81** linear morphometric variation of leg I length between males and females of *L. tenochtitlan* and *L. misteca* (T test) (males: t = 3.6174, p = 0.00331, df = 13, α = 0.05; females: t = 0.274, p = 0.787, df = 17, α = 0.05).

The analysis of canonical variables CVA shows a significant difference (χ^2^ = 10.2555, df = 2, p = 0.00593003, λ = 0.5988) between both species, which indicates the formation of two groups according to the tibiae shape of the palps of the males (Fig. 74). The differences on the tibiae can be observed in the deformation rack, where a deformation is shown mainly in the ventral-basal and the dorsal-apical parts (Fig. 75). In this way, the tibiae of *L. tenochtitlan* sp. nov. is thinner in ventral-basal part (Fig. 77), whereas in *L. misteca* the ventral-basal part is wider and slightly less curved in the dorsal-apical part (Fig. 76). To analyze sexual dimorphism and variation in the new species, a T-test showed that between the males and females of *L. tenochtitlan* sp. nov., there are no statistically significant differences in leg I length (t = -1.3106, p = 0.1981, df = 37, α = 0.05), carapace length (t = 1.498, p = 0.142, df = 38, α = 0.05), and carapace width (t = 0.6955, p = 0.4912, df = 3*6*, α = 0.05) (Figs 78–80). Therefore, there is no secondary sexual dimorphism between males and females of the new species (Table 5, Figs 78–81). However, a T-test showed that there is secondary sexual dimorphism between males and females of *L. misteca* in leg I length (t = 3.1086, p = 0.0038, df = 21, α = 0.05) (Fig. 81). A T-test indicated that there are statistically significant differences between the new species and *L. misteca* in leg I length of males (t = 3.6174, p = 0.00331, df = 13, α = 0.05) with the longest legs occurring in *L. misteca* (Table 5, Fig. 81). There was no statistical support for significant differences in leg I length between females of each species (t = 0.274, p = 0.787, df = 17, α = 0.05) (Table 5, Fig. 81).

**Table 5.** Average of linear measurements of *Loxosceles tenochtitlan* sp. nov. and *Loxosceles misteca*. *N* = number of individuals. LL1 = Length of leg 1. Cl = Carapace length. Cw = Carapace width. Sl = Sternum length. Sw = Sternum width. ♂ = males. ♀ = females. Numbers in parentheses represent minimum and maximum measurements.

Species	*N*	LL1	Cl	Cw	Sl	Sw
*Loxosceles tenochtitlan* sp. nov.	♂ 16	18.10	3.00	2.70	1.80	1.50
		(13.8–21.3)	(2.2–4.2)	(2.2–3.2)	(1.6–2.1)	(1.2–2.0)
	♀ 24	22.36	2.94	2.71	1.55	1.38
		(17.7–26.5)	(1.8–3.9)	(2.2–3.1)	(1.3–2.1)	(1.2–1.9)
*Loxosceles misteca*	♂ 11	23.75	3.05	2.73	1.59	1.42
		(18–31.9)	(2.5–3.4)	(2.5–3.0)	(1.2–1.9)	(1.1–1.5)
	♀ 11	18.47	3.08	2.67	1.59	1.31
		(14.1–18.9)	(2.5–3.3)	(2.3–3.0)	(1.4–1.9)	(1.1–1.7)

### Ecological niche modeling (ENM)

**Figures 84.**
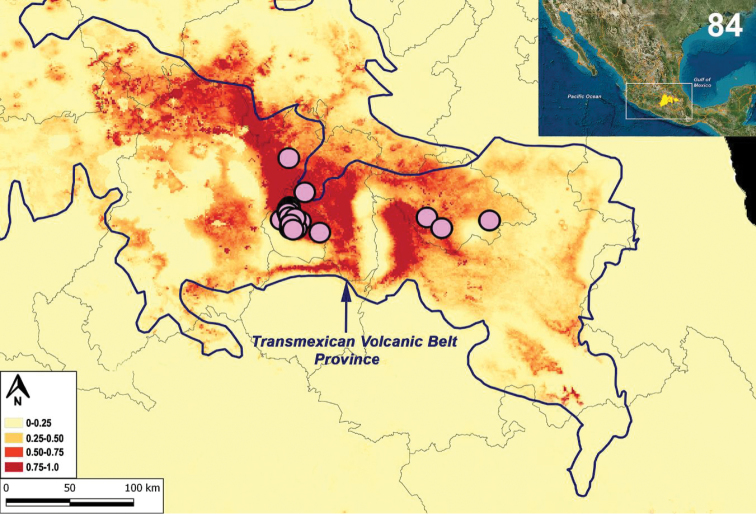
Ecological niche modeling (ENM) under Maxent algorithm for *Loxosceles tenochtitlan* sp. nov. Colors represent different ranges of probabilities of presence (high probability: 0.75–1.0). Circles represent known records of the new species. Blue lines represent biogeographical provinces proposed by Morrone (2004, 2005).

To analyze the potential distribution of *L. tenochtitlan* sp. nov., ENM was performed for the new species, with a total of 34 records from Mexico City, Estado de Mexico and Tlaxcala (Figs 82–84). The highest contribution to the model came from Vegetation Type (CON01) with 42% and Mean Temperature of Wettest Quarter (BIO10) with 28.5% (Table 6). Additionally, the Area Under the Curve (AUC) demonstrated good performance AUC= 0.993.

**Table 6.** Percent contribution of the climatic variables for the distribution model for *Loxosceles tenochtitlan* sp. nov. using the Maxent algorithm.

Variables	Contribution (%)
Vegetation type (CON01)	42
Mean Temperature of Wettest Quarter (BIO10)	28.5
Max Temperature of Warmest Month (BIO05)	7.2
Temperature Seasonality (BIO04)	5.3

**Figure﻿ 82–83.**
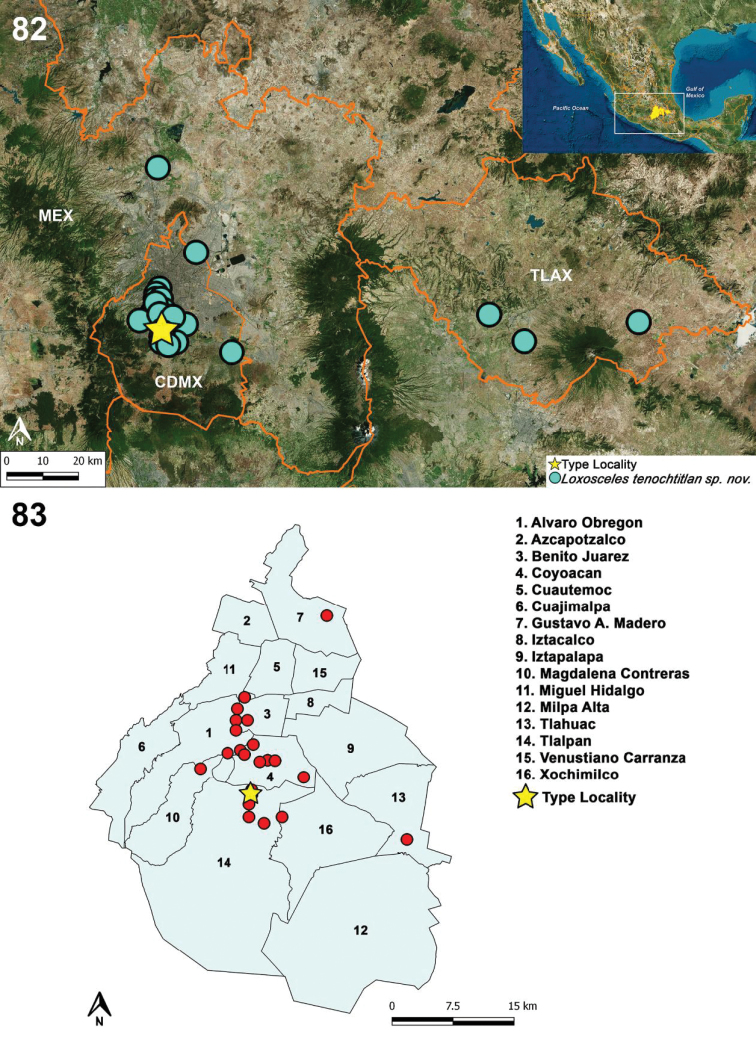
**82** Distribution records of *Loxosceles tenochtitlan* sp. nov. from Mexico City (CDMX), Estado de Mexico (MEX), and Tlaxcala (TLAX) **83** known records of *L. tenochtitlan* sp. nov. from Mexico City, including the type locality (star).

Following the biogeographic scheme for Mexico proposed by Morrone (2004, 2005), the highest probability of the presence of *L. tenochtitlan* sp. nov. (0.75–1.0) was markedly toward the biogeographical province of the Transmexican Volcanic Belt (TVB), with a potential distribution including Mexico City, north of Estado de Mexico, west of Puebla, most of Tlaxcala, and a small portion of Hidalgo and Queretaro (Fig. 84).

## Discussion

The first record of *Loxosceles* from Mexico City was by Gertsch (1958), who reported a female of *Loxosceles nahuana* Gertsch, 1958, a native species from Zimapán, Hidalgo; however, this record is a misidentification because posteriorly Gertsch and Ennik (1983) did not consider this record in their taxonomic revision of *Loxosceles* from North America. Hoffmann (1976) includes the same record of *L. nahuana* in her preliminary list of Mexican spiders, but she did not mention other species. Francke et al. (2009), Durán-Barrón and Pérez-Ortíz (2016) and Durán-Barrón and Ayala-Islas (2007) reported two species from Mexico City, *L. misteca* and one unidentified species of *Loxosceles*, comprising a single female, two males and two immature specimens. Surprisingly, the authors never identified it to species level. Unfortunately, we did not have access to those collections; therefore, we do not know whether there are two species or only one from Mexico City. In this way, *L. nahuana* is a valid and different species as the species delimitation methods and different topologies showed (Figs 70–72), even this species is not closely related with the new species described herein neither with *L. misteca* (Figs 70–72). In the present work, all the specimens reviewed belong to *Loxosceles tenochtitlan* sp. nov., therefore we can assume that the previous records of *L. misteca* belong to the new species described herein, and that *L. misteca* is not found in Mexico City or the rest of the states where the new species has been recorded (Estado de Mexico and Tlaxcala). Recently, Valdez-Mondragón et al. (2018a, b) mentioned that *L. misteca* from Mexico City and Tlaxcala was an introduced species, however this was an incorrect interpretation. *Loxosceles misteca* is a species from Guerrero and Morelos, whereas the records of *L. misteca* from Mexico City and Tlaxcala belong to *L. tenochtitlan* sp. nov., a native species of the region (Fig. 82–84). Only two introduced species have been recorded in Mexico, *Loxosceles reclusa* Gertsch & Mulaik, 1940 from the south-central United States and *Loxosceles rufescens* (Dufour, 1820), a widely distributed species throughout the Mediterranean Basin and the Middle East (Gertsch 1958, 1973; Gertsch and Ennik 1983; Nentwig et al. 2017; Tahami et al. 2017; Valdez-Mondragón et al. 2018a, b; WSC 2019).

As was mentioned previously, recent taxonomic studies based on molecular analyses using mitochondrial markers have suggested that the known diversity within the genus *Loxosceles* could be greatly underestimated (Binford et al. 2008; Duncan et al. 2010; Planas and Ribera 2014, 2015; Tahami et al. 2017). Additionally, it has been decades since a revision of the North American species has been conducted, and given the intraspecific variation in sexual structures, primarily in the seminal receptacles in the females (Brignoli 1968, Gertsch and Ennik 1983) this can be very difficult. Despite this, the male palps remain a good character for species identification because there is little morphological variation in comparison with seminal receptacles as was showed by Valdez-Mondragón et al. (2018b) recently in the description of *Loxosceles malintzi*.

Although DNA barcodes are being applied in modern systematics as a useful tool to resolve species delimitation problems, modern taxonomy includes many different sources of evidence, such as traditional morphology, ecology, reproduction, and biogeography. Traditional morphology alone cannot determine species boundaries in some cases, and the genus *Loxosceles* is no exception. Identifying morphologically inseparable cryptic or sibling species requires a new set of taxonomic tools, including DNA and additional sources of evidence (integrative taxonomy) (Jarman and Elliott 2000; Witt and Hebert 2000; DeSalle et al. 2005; Hebert et al. 2003, 2004; Bickford et al. 2007; Hamilton et al. 2011, 2014, 2016; Ortiz and Francke 2016). The researchers should apply different range of species delimitation method at the same time to their data and place their truth in delimitation that are congruent across methods (Carstens et al. 2013). Using several species delimitation methods, incongruence across the different results is evidence of either a difference in the power to detect cryptic lineages across one or more of the approaches used to delimit species and could indicate that assumptions of one or more of the methods have been violated, in this cases the assumptions for species delimitations should be conservative (Carstens et al. 2013). In this work, the four different molecular species delimitation methods were congruent and consistent to separate *L. tenochtitlan* sp. nov and *L. misteca* (Fig. 72).

Although morphologically *L. tenochtitlan* sp. nov is quite similar to *L. misteca* in the seminal receptacles of the females and the male palps, there are some subtle morphological differences that allow diagnosis of the new species as was mentioned in the description section. Multiple lines of robust evidence are able to clearly separate it as a new species. These methods are genetic differences, geometric and linear morphometry and different biogeographical distribution patterns. Strictly, cryptic species are those that cannot be differentiated based on their morphology or external appearance and are reproductively isolated. The present genetic divergence indicates the two species are independent lineages (Bickford et al. 2007; Hebert et al. 2004; Struck and Cerca 2019).

The species separation based on corrected genetic distances indicates that CO1 performed better for species delimitation than ITS2 (Figs 70, 71). This result confirms the utility of DNA barcoding as a fast and reliable tool for the identification and species delimitation of the *Loxosceles* from the *reclusa* group of North America. Similar results have also been found in other molecular studies of *Loxosceles*. Planas and Ribera (2014, 2015) found genetic distances between species from the Canary Islands to be > 12% using COI, whereas Tahami et al. (2017) found genetic distances between species from the Middle East ranged for CO1 from 17.5 to 20.6%. Additionally, CO1 haplotypes network also corroborated the distinctiveness of the different species (Fig. 73). The approaches for analyzing DNA barcode data, using *p*-distances for CO1 and ITS2 and tree-based delimitation with ML and BI (CO1+ITS2), recovered a monophyletic cluster with high support values for the samples of *L. tenochtitlan* sp. nov from Mexico City + Tlaxcala (Figs 70–72), as well as another monophyletic cluster of the samples of *L. misteca* from Guerrero, where some samples were collected near the type locality of the species as well as localities previously reported by Gertsch (1958) and Gertsch and Ennik (1983) (Figs 41, 62–69).

Sexual characters in spiders are robust and important morphological characters that are still used to separate species and to provide a diagnosis. This means that genitalia evolve, on average, more rapidly than non-genital morphological traits (Huber, 2003; Huber and Dimitrov 2014). Also, the somatic characters are useful as additional evidence to separate species in some groups of spiders; coloration, color pattern, body proportions, and even extreme size differences are useful traits for species separation (Huber et al. 2005; Huber and Dimitrov 2014). As additional evidence for the separation between *L. tenochtitlan* sp. nov. and *L. misteca*, geometric and linear morphometric variation was statistically significant for tibia shape of the palp of males and leg I length between males of both species, where the males of *L. misteca* have longer legs than the males of the new species (Table 5, Fig. 81). We do not know whether these differences in leg lengths between males of both species correspond to the microhabitat of each species or why this morphological difference only occurs in males. *Loxosceles tenochtitlan* sp. nov. only has been collected in urban areas (Figs 7–18), whereas *L. misteca* are common in caves and have been collected from caves in Guerrero and Estado de Mexico. Some studies have demonstrated how microhabitat plays an important role in driving spider diversification. Eberle et al. (2018) analyzed diversification in pholcids based on the framework of the largest molecular phylogeny of the spider family Pholcidae to date, analyzed their diversification and found that diversity may be caused by microhabitat changes. Planas and Ribera (2014) and Souza and Ferreira (2018) mentioned that *Loxosceles* are generally considered troglophiles because of their abundance in caves. In other animals, long legs are considered a hallmark of troglomorphism. Further research of North American species of *Loxosceles* is required to address a correlation between leg length and microhabitat.

ENM is a powerful approach to understand how abiotic factors (e.g., temperature, precipitation, and seasonality) impact the geographic limits of the species (Graham et al. 2004a; Wiens and Graham 2005). The integration of genetic and ecological approaches in the study of mechanisms driving geographic distributions of organisms is becoming more common (Hugall et al. 2002; Johnson and Cicero 2002; Graham et al. 2004; Lapointe and Rissler 2005; Rissler and Apodaca 2007; Raxworthy et al. 2007). In the ENM, following the biogeographical provinces proposed by Morrone (2004, 2005), vegetation type plays an important role in the ecological niche of the species (Fig. 84). ENM showed that the highest probability of presence (0.75–1.0) for *L. tenochtitlan* sp. nov. is strongly limited towards the Transmexican Volcanic Belt (TVB) (Fig. 84), characterized by high mountains and a temperate climate, with pine, oak or oak-pine forest. Although ENM calculated a potential distribution to the south of states of Puebla, south and north of the Estado de Mexico, and small regions of the states of Michoacan, Guanajuato and Queretaro, this can be explained as an over-prediction, and other species of *Loxosceles* might occur there (Fig. 84) (Valdez-Mondragón et al. 2018: figs 75–77). Although *L. tenochtitlan* sp. nov. is distributed widely in urban areas of Mexico City, Estado de Mexico and Tlaxcala, this species can be considered a native of this region and the urbanization process has not affected its establishment in such areas. However, the species has never been collected in natural areas in the state (Valdez-Mondragón et al. 2018a, b). In 2017, four collectors collected around 40 specimens of *L. tenochtitlan* sp. nov. in two hours from a house in the state of Tlaxcala, Mexico (Valdez-Mondragón et al. 2018a, b). As has been demonstrated for other species of the genus as *Loxosceles reclusa* from the United States, the partial synanthropy of some species of the brown recluse spiders is not the dominant influence on distributional patterns (Saupe et al. 2011). Although the species may be able to expand beyond their distribution with the aid of the anthropogenic activities, the species analyzed herein does not have widespread distribution due to historical or biological barriers or their limited dispersion potential, where the vegetation type plays an important role to delimitation of their distribution (Table 6, Fig. 84).

Despite the similarity between *L. tenochtitlan* sp. nov. and *L. misteca*, we consider them different species for three main reasons: (1) they can be distinguished by morphological characters (genitalic and somatic); and the new species can be diagnosed morphologically; (2) molecular data from multiple genes analyzed with multiple methods consistently separate them (congruence among methods); and (3) statistically significant geometric and linear morphometric variation in tibias shape of the palp of the male and leg I length of males respectively.

## Supplementary Material

XML Treatment for Loxosceles

XML Treatment for Loxosceles tenochtitlan

XML Treatment for Loxosceles misteca
